# Systems prediction of chronic lung allograft dysfunction: Results and perspectives from the Cohort of Lung Transplantation and Systems prediction of Chronic Lung Allograft Dysfunction cohorts

**DOI:** 10.3389/fmed.2023.1126697

**Published:** 2023-03-09

**Authors:** Christophe Pison, Adrien Tissot, Eric Bernasconi, Pierre-Joseph Royer, Antoine Roux, Angela Koutsokera, Benjamin Coiffard, Benjamin Renaud-Picard, Jérôme Le Pavec, Pierre Mordant, Xavier Demant, Thomas Villeneuve, Jean-Francois Mornex, Simona Nemska, Nelly Frossard, Olivier Brugière, Valérie Siroux, Benjamin J. Marsland, Aurore Foureau, Karine Botturi, Eugenie Durand, Johann Pellet, Richard Danger, Charles Auffray, Sophie Brouard, Laurent Nicod, Antoine Magnan, Members of the Cohort of Lung Transplantation and Systems prediction of Chronic Lung Allograft Dysfunction consortia

**Affiliations:** ^1^Service Hospitalier Universitaire de Pneumologie Physiologie, Pôle Thorax et Vaisseaux, Fédération Grenoble Transplantation, CHU Grenoble Alpes, Grenoble, France; ^2^Université Grenoble Alpes, INSERM 1055, Grenoble, France; ^3^Service de Pneumologie, Institut du Thorax, CHU Nantes, Nantes, France; ^4^CHU Nantes, Nantes Université, INSERM, Center for Research in Transplantation and Translational Immunology (CR2TI), UMR 1064, ITUN, Nantes, France; ^5^Unité de Transplantation Pulmonaire, Service de Pneumologie, Centre Hospitalier Universitaire Vaudois et Université de Lausanne, Lausanne, Suisse; ^6^Service de Pneumologie, Hôpital Foch, Suresnes, France; ^7^Institut National de Recherche Pour l’Agriculture, l’Alimentation et l’Environnement, INRAE, Jouy-en-Josas, France; ^8^Service de Pneumologie et de Transplantation Pulmonaire, APHM, Hôpital Nord, Aix Marseille Univ, Marseille, France; ^9^Service de Pneumologie, Hôpitaux Universitaires de Strasbourg, Strasbourg, France; ^10^Inserm UMR 1260, Regenerative Nanomedicine, Université de Strasbourg, Strasbourg, France; ^11^Service de Chirurgie Thoracique, Vasculaire et Transplantation Cardiopulmonaire, Centre Chirurgical Marie Lannelongue, Le Plessis Robinson, France; ^12^Service de Chirurgie Vasculaire, Thoracique et Transplantation Pulmonaire, Hôpital Bichat, AP-HP, INSERM U1152, Université Paris Cité, Paris, France; ^13^Service de Pneumologie et Transplantation Pulmonaire, CHU de Bordeaux, Bordeaux, France; ^14^Service de Pneumologie, CHU de Toulouse, Université Toulouse III-Paul Sabatier, Toulouse, France; ^15^Université de Lyon, Université Lyon 1, PSL, EPHE, INRAE, IVPC, Lyon, France; ^16^Hospices Civils de Lyon, GHE, Service de Pneumologie, RESPIFIL, Orphalung, Inserm CIC, Lyon, France; ^17^UMR 7200 - Laboratoire d'Innovation Thérapeutique, Faculté de Pharmacie, CNRS-Université de Strasbourg, Illkirch, France; ^18^Service de Pneumologie, Hôpital Foch, Suresnes, France; ^19^Laboratoire d’Immunologie de la Transplantation, Hôpital Saint-Louis, CEA/DRF/Institut de Biologie François Jacob, Unité INSERM 1152, Université Paris Diderot, USPC, Paris, France; ^20^Team of Environmental Epidemiology Applied to the Development and Respiratory Health, Institute for Advanced Biosciences (IAB), Inserm U1209, CNRS UMR 5309, Université Grenoble Alpes, Grenoble, France; ^21^Department of Immunology and Pathology, Central Clinical School, Monash University, Melbourne, VIC, Australia; ^22^European Institute for Systems Biology and Medicine, Vourles, France

**Keywords:** chronic lung allograft dysfunction, bronchiolitis obliterans syndrome, restrictive allograft syndrome, chronic rejection, Frontiers in medicine, pulmonary section

## Abstract

**Background:**

Chronic lung allograft dysfunction (CLAD) is the leading cause of poor long-term survival after lung transplantation (LT). Systems prediction of Chronic Lung Allograft Dysfunction (SysCLAD) aimed to predict CLAD.

**Methods:**

To predict CLAD, we investigated the clinicome of patients with LT; the exposome through assessment of airway microbiota in bronchoalveolar lavage cells and air pollution studies; the immunome with works on activation of dendritic cells, the role of T cells to promote the secretion of matrix metalloproteinase-9, and subpopulations of T and B cells; genome polymorphisms; blood transcriptome; plasma proteome studies and assessment of MSK1 expression.

**Results:**

Clinicome: the best multivariate logistic regression analysis model for early-onset CLAD in 422 LT eligible patients generated a ROC curve with an area under the curve of 0.77. Exposome: chronic exposure to air pollutants appears deleterious on lung function levels in LT recipients (LTRs), might be modified by macrolides, and increases mortality. Our findings established a link between the lung microbial ecosystem, human lung function, and clinical stability post-transplant. Immunome: a decreased expression of CLEC1A in human lung transplants is predictive of the development of chronic rejection and associated with a higher level of interleukin 17A; Immune cells support airway remodeling through the production of plasma MMP-9 levels, a potential predictive biomarker of CLAD. Blood CD9-expressing B cells appear to favor the maintenance of long-term stable graft function and are a potential new predictive biomarker of BOS-free survival. An early increase of blood CD4 + CD57 + ILT2+ T cells after LT may be associated with CLAD onset. Genome: Donor Club cell secretory protein G38A polymorphism is associated with a decreased risk of severe primary graft dysfunction after LT. Transcriptome: blood POU class 2 associating factor 1, T-cell leukemia/lymphoma domain, and B cell lymphocytes, were validated as predictive biomarkers of CLAD phenotypes more than 6 months before diagnosis. Proteome: blood A2MG is an independent predictor of CLAD, and MSK1 kinase overexpression is either a marker or a potential therapeutic target in CLAD.

**Conclusion:**

Systems prediction of Chronic Lung Allograft Dysfunction generated multiple fingerprints that enabled the development of predictors of CLAD. These results open the way to the integration of these fingerprints into a predictive handprint.

## Introduction

Lung transplantation (LT) is an accepted and validated treatment for end stage respiratory diseases in selected patients despite the lowest median long-term survival among solid organ transplantations (1, 2). Chronic lung allograft dysfunction (CLAD) is the leading cause of poor long-term survival after LT accounting for more than 40% of deaths beyond the first years (2). Therefore, CLAD is the main limitation to achieving optimal medium and long-term survivals after LT. Our hypothesis was that a systems prediction approach for CLAD could pave the way to preventive and/or early interventions (3, 4).

Two prospective multi-centric cohorts of LT, Cohort of Lung Transplantation (COLT) and Systems prediction of Chronic Lung Allograft Dysfunction (SysCLAD) aimed to tackle this ambitious objective, i.e., prediction of CLAD after LT (3, 5).

The first step achieved at the international level in 2019 was the adoption of a CLAD definition with two landmark consensus documents of the International Society for Heart and Lung Transplantation (ISHLT) (4, 6), updated in 2020 with new data (7). CLAD refers to the persistent decline of 20% over 3 months of the forced expiratory volume in one second (FEV_1_) and/or the forced vital capacity (FVC) that cannot be attributed to a specific cause other than chronic graft rejection (4). Bronchiolitis obliterans syndrome (BOS) and restrictive allograft syndrome (RAS) are considered the two main phenotypes of CLAD, BOS being much more prevalent than RAS representing, respectively, 60 and 15% of CLAD in a 2020 retrospective mono-centric study (4, 6, 7). A mixed CLAD phenotype with restrictive and obstructive defects has also been described (8) or other combinations such as obstructive defects with persistent opacities (7). Diagnosis of BOS or RAS requires the exclusion of alternative diagnoses, which may be challenging in explaining in part why 25% of CLAD cases remain either unclassified or undefined (7, 9).

Bronchiolitis obliterans syndrome is characterized by persistent airflow obstruction in the absence of a restrictive ventilation defect and imaging studies that may be unremarkable or show air trapping (4, 7). RAS is characterized by a restrictive ventilation defect and radiological signs of fibrosis or infiltrates (4, 6, 7). Prognosis is worse for RAS, with a mean time to death of 372 days compared to 500 days in BOS (4, 6, 7). For RAS, the time of diagnosis after transplant does not seem to influence survival.

Although the pathophysiological mechanisms and the risk factors implicated in BOS and RAS are far from being fully elucidated, literature provides increasing evidence and novel insights, especially regarding auto-antibodies (4, 6, 10, 11). Identification of risk factors associated with specific CLAD phenotypes together with relevant biomarkers (12, 13) is of crucial importance as it may assist patient risk stratification, optimize follow-up, and allow prevention and early intervention for potentially modifiable factors. In the field of kidney transplantation, this objective has been reached in 2021 by an international consortium (14). Prediction of CLAD was the main objective of the COLT and SysCLAD cohorts for which we report here the most significant results obtained so far.

## Patients and methods

The cohort of lung transplantation, NCT00980967 was initiated on September 2009 with the inclusion of the first patient whereas the last one was recruited by January 2019. The study was designed in the context of merging efforts of working groups of the French-speaking Pulmonary Society, the French-speaking Society of Transplantation, and the French Society of Thoracic and Cardiovascular Surgery. The design of COLT began at the end of 2007 with the wish of all French centers to carry out a multicenter project in order to unravel predictive factors for CLAD of which only bronchiolitis obliterans (BOS) was the known clinical form at that time (5). The main objective of COLT was the discovery of early factors able to predict CLAD. The method was to set a prospective cohort of patients included before the transplant, then regularly followed over a period of 10 years of inclusion. Every 6 months after the initial visit and up to 5 years of patient follow-up, clinical, physiological, radiologic, and biological data were collected and stored. By February 2021, COLT had included 1874 patients and among them, 1,618 have been transplanted; of these, 1,106 (68.4%) could be followed up over 3 years, 905 (55.9%) over 5 years, and 117 (7.2%) over 10 years.

The SysCLAD cohort (3) was created thanks to significant efforts to merge two multi-centric cohorts already constituted, different in their structures but able to interact, COLT (5) on the one hand and the Swiss Transplant Cohort Study (STCS) (15, 16) on the other hand. The two Swiss LT centers, Zurich and Lausanne/Geneva, participated in STCS since its establishment in April 2008. By February 2012, 282 LT were included in STCS. SysCLAD was a short 2-year pilot European project in systems medicine for which the inclusion period was reduced (3). This project proposed transplantation as a demonstration of the feasibility of implementing a systems medicine approach to address an unmet medical need, i.e., to predict CLAD after LT. In turn, SysCLAD attracted laboratories in France and Switzerland recognized for their expertise in immunology (INSERM 1064 in Nantes, INSERM 1087 in Nantes, INSERM 1152 in Paris, CNRS/UdS 7200 in Strasbourg), in epidemiology (INSERM U1209/CNRS 5309 in Grenoble), respiratory microbiota (Centre Hospitalier Universitaire Vaudois - Université in Lausanne) and systems analysis (European Institute for Systems Biology and Medicine-EISBM in Lyon).

Lung transplantation recipients were regularly assessed for CLAD thanks to adjudication committees accordingly to 2014 ISHLT (17) recommendations updated in 2019–2020 (5, 8, 9). This committee of LT specialists from participating centers evaluated pulmonary function tests (PFTs), imaging studies, and confounding factors to identify LTR who remained stable or developed a definite BOS or RAS. Actions to blind assessment included data anonymization and an initial evaluation of the PFTs before the assessment of imaging studies and confounding factors.

## Results

There were 15 original works published by January 2022 within 13 years devoted to the prediction of CLAD (18–32) besides a protocol (3), reviews (5, 12, 33), or collateral work (34). These studies were related, respectively, to 1-“clinicome” to calibrate a multivariate model for early-onset BOS and RAS (23), 2-“exposome” with airway microbiota (18, 21, 32) and air pollution studies (20), 3-“immunome” with works on activation of dendritic cells (19), role of T cells in promoting the secretion of matrix metaloproteinase-9 (22, 24), subpopulations of T and B cells to predict BOS (26, 27, 31), 4-“genome studies” including polymorphism of Club cell secretory protein related to primary allograft dysfunction (29), 5-“transcriptome and proteome” with the signature of CLAD (25), with the role of plasma acute phase protein to predict CLAD (28) and the progressive overexpression of MSK1 kinase on transbronchial biopsies (TBB) in patients with CLAD (30).

### Clinicome

#### Calibration of a multivariate model for early-onset bronchiolitis obliterans syndrome and restrictive allograft syndrome

**Figure 1:**
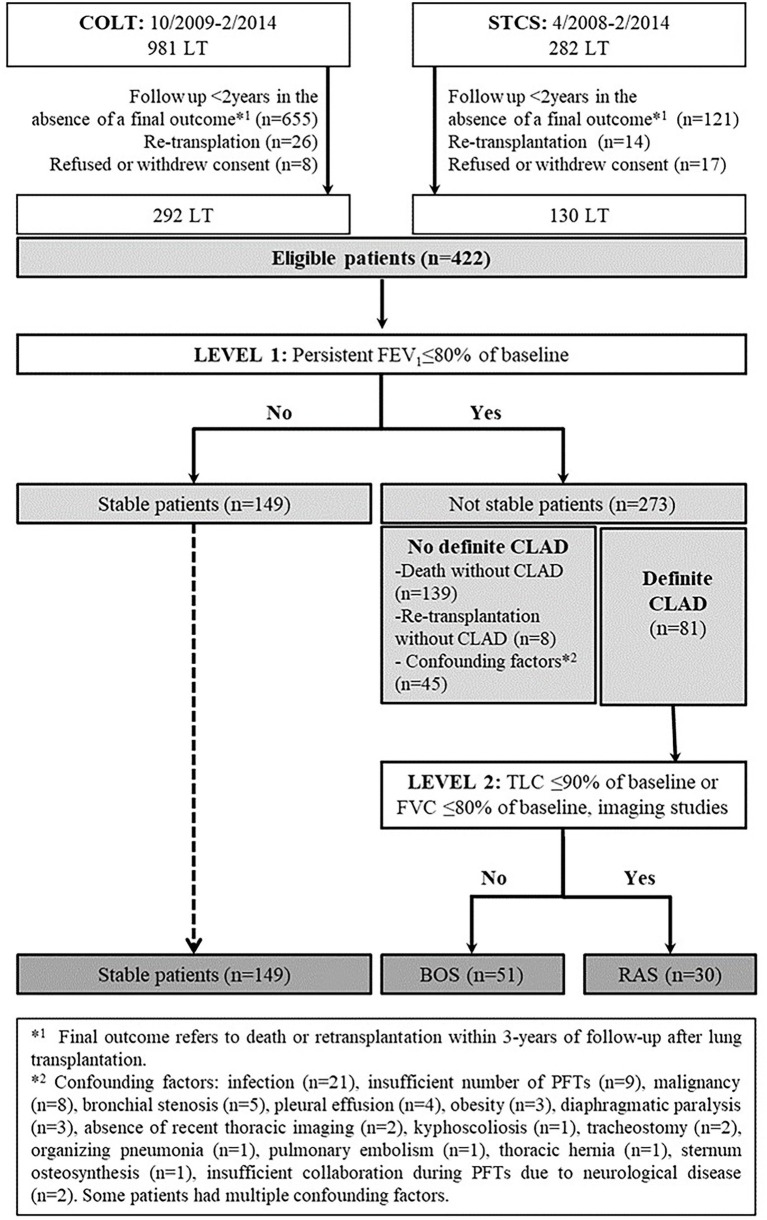
Flow chart diagram of the evaluated population, adapted from (23).

Lung transplantation recipients of the COLT and STCS were eligible for inclusion in the SysCLAD cohort when alive with a follow-up period of at least 2 years but less than 3 years, or if they died or were retransplanted within 3 years (23). An adjudication committee assessed these patients for early-onset BOS, RAS, or stable allograft function. Baseline characteristics, data on surgery, immunosuppression, and year-1 follow-up were collected. Prediction models for BOS and RAS within 3 years post-LT were developed using multivariate logistic regression and multivariate multinomial analysis. Among those fulfilling the eligibility criteria (292 and 130 from COLT and STCS, respectively, Figure 1), we identified 149 stable, 51 BOS, and 30 RAS LT patients. The variables of the best performing multivariate logistic regression analysis model to predict early-onset CLAD included recipient age, underlying diagnosis, induction treatment, and presence of year-1 class II donor-specific antibodies (DSAs). The prediction model generated a ROC curve with an area under the curve (AUC) of 0.77. The ROC curve of the same model but without using year-1 class II DSAs provided an AUC of 0.73. The best prediction model for early-onset BOS and RAS included recipient age, the underlying diagnosis, induction treatment with rabbit anti-thymocyte globulin (rATG), immunosuppression with tacrolimus, and year-1 class II DSAs. Within this model, class II DSAs were associated with BOS and RAS, whereas pre-LT diagnoses of interstitial lung disease and chronic obstructive pulmonary disease were in addition associated with RAS (Table 1).

**Table 1:** Risk factors for BOS and RAS by 3 years post-LT as compared to stable recipients by multivariate multinomial analysis, adapted from (23).

Variable		BOS		RAS	
		OR (95% CI)	*p* value	OR (95% CI)	*p* value
Underlying diag.	*CF*	baseline		baseline	
	COPD	1.61 (0.56, 4.61)	0.38	3.86 (1.04, 14.29)	**0.04**
	ILD/IPF	2.44 (0.74, 8.04)	0.14	5.47 (1.48, 20.17)	**0.01**
	Other	2.589 (0.85, 7.91)	0.09	0.23 (0.02, 2.40)	0.22
Immunosuppression	Cyclosporin	baseline		baseline	
	Tacrolimus	3.18 (0.70, 14.36)	0.13	0.67 (0.08, 5.59)	0.71
Induction treatment	Basiliximab	baseline		baseline	
	None	0.54 (0.12, 2.51)	0.43	4.53 (0.89, 23.08)	0.07
	rATG	3.10 (0.68, 14.12)	0.14	2.39 (0.31, 18.67)	0.41
Y1 DSAs II	Yes	3.83 (1.46, 10.04)	**0.006**	6.97 (1.84, 26.38)	**0.004**

Results are presented as odds-ratio (95% confidence intervals). For binary variables, the ‘No’ group has been considered the baseline group. CF, cystic fibrosis; COPD, chronic obstructive pulmonary disease; DSAs, donor specific antibodies; ILD/IPF, interstitial lung disease/idiopathic pulmonary fibrosis; rATG, rabbit antithymocyte globulin; t, treated; Y1, year 1.

### Exposome

#### Chronic effect of pollution

**Figure 2:**
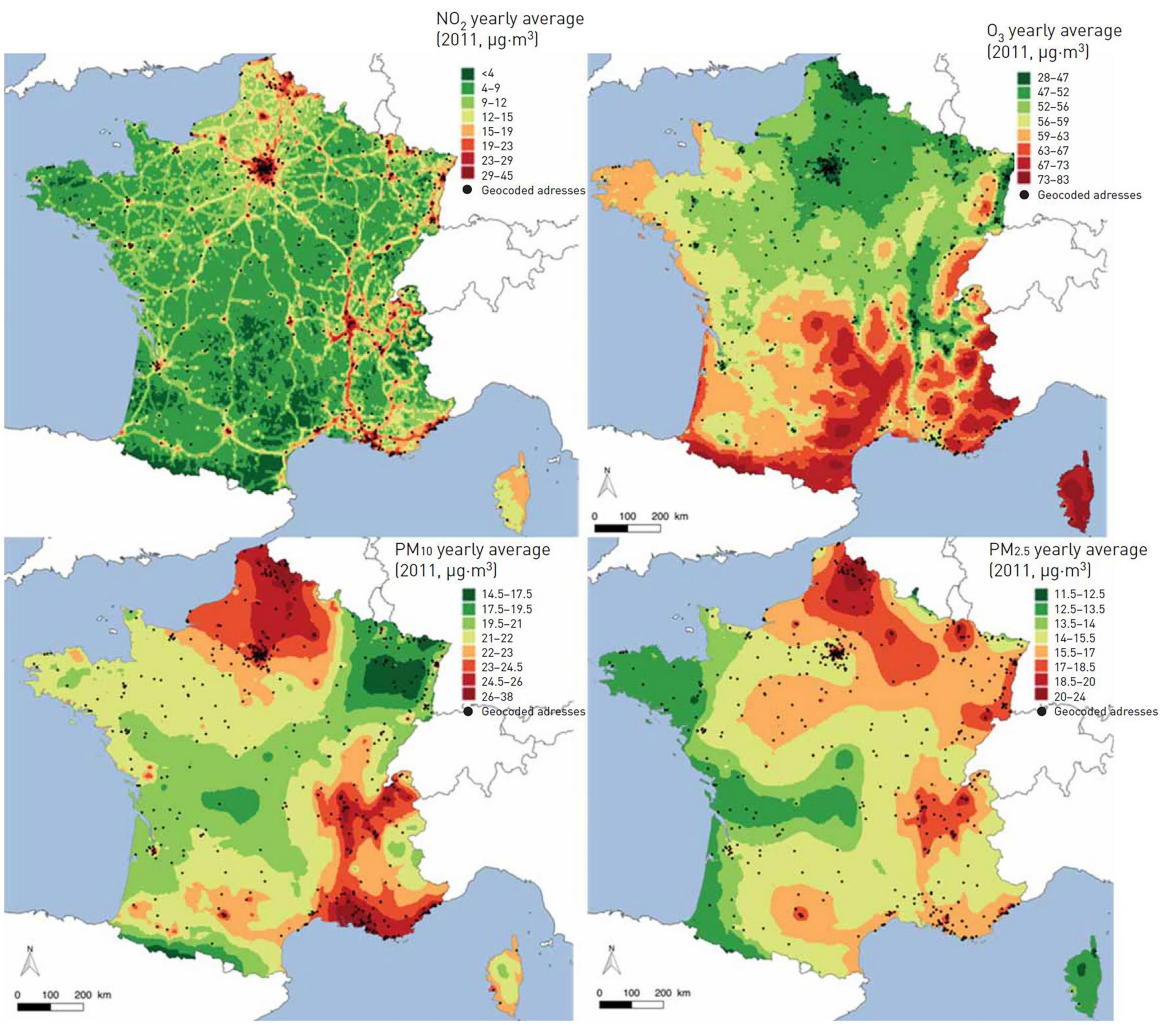
Averaged 12-month concentration of particulate matter with an aerodynamic cut-off of 2.5 μm (PM2.5) and 10 μm (PM10), NO2 and O3 across France in 2011, adapted from (20).

**Figure 3:**
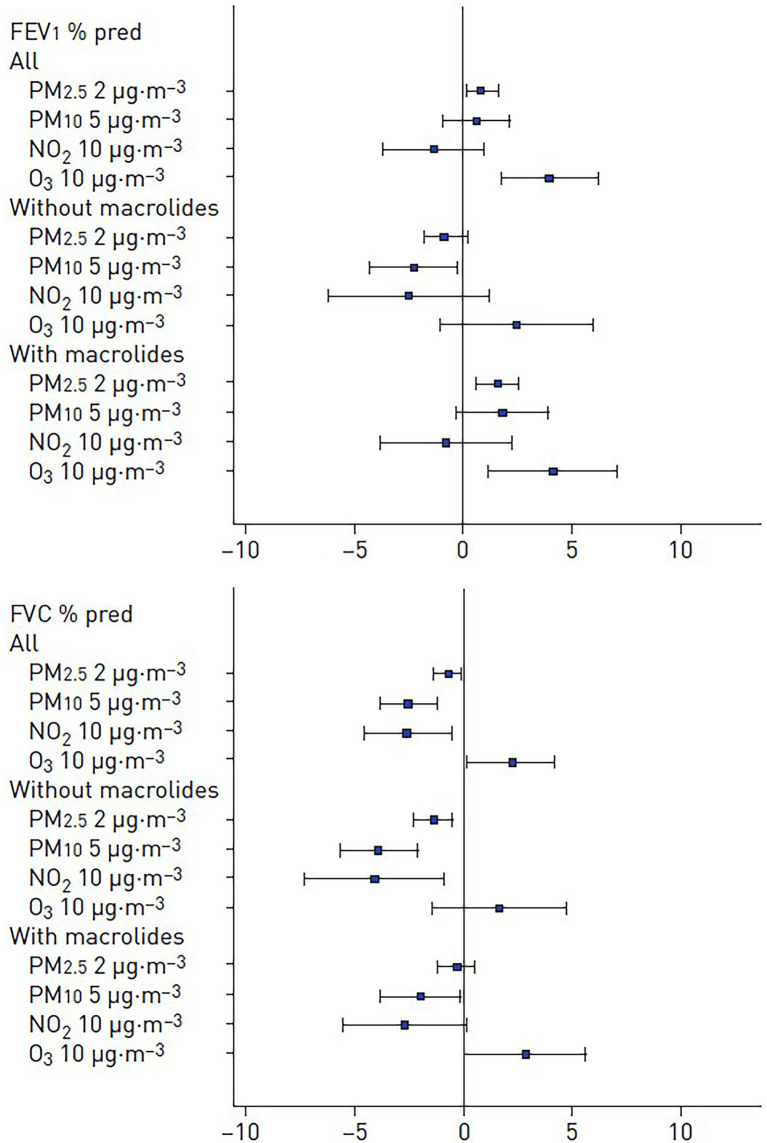
Adjusted associations between air pollutants exposure and level of **(A)** FEV_1_% predicted and **(B)** FVC% predicted in the whole population and according to the use of macrolides. PMx: particulate matter with an aerodynamic cross section of x μm, adapted from (20).

The role of chronic exposure to ambient air pollution on lung function levels in LTRs was evaluated (20). The lung function of 520 LTRs from the COLT study was measured every 6 months post-LT. The levels of air pollutants [nitrogen dioxide (NO2)], particulate matter with an aerodynamic cut-off diameter of x μm (PMx), and ozone (O3) at the patients’ home were averaged in the 12 months before each spirometry test, Figure 2. The effects of air pollutants on FEV_1_ and FVC in % predicted were estimated using mixed linear regressions. We assessed the modification effect of macrolide antibiotics in this relationship. Increased 12-month levels of pollutants were associated with lower levels of FVC% pred. −2.56, 95% CI −3.86−−1.25 for 5 μg·m^3^ of PM10; −0.75, 95% CI −1.38−−0.12 for 2 μg·m^3^ of PM2.5 and − 2.58, 95% CI −4.63−−0.53 for 10 μg·m^3^ of NO2, Figure 3. In patients not taking macrolides, the deleterious association between PM and FVC tended to be stronger and PM10 was associated with lower FEV_1_, Figure 3. This study suggests a deleterious effect of chronic exposure to air pollutants on lung function levels in LTRs, which might be modified by macrolides. The results of a complementary analysis *in progress* indicate that air pollution increases mortality.

#### Airway microbiota

**Figure 4:**
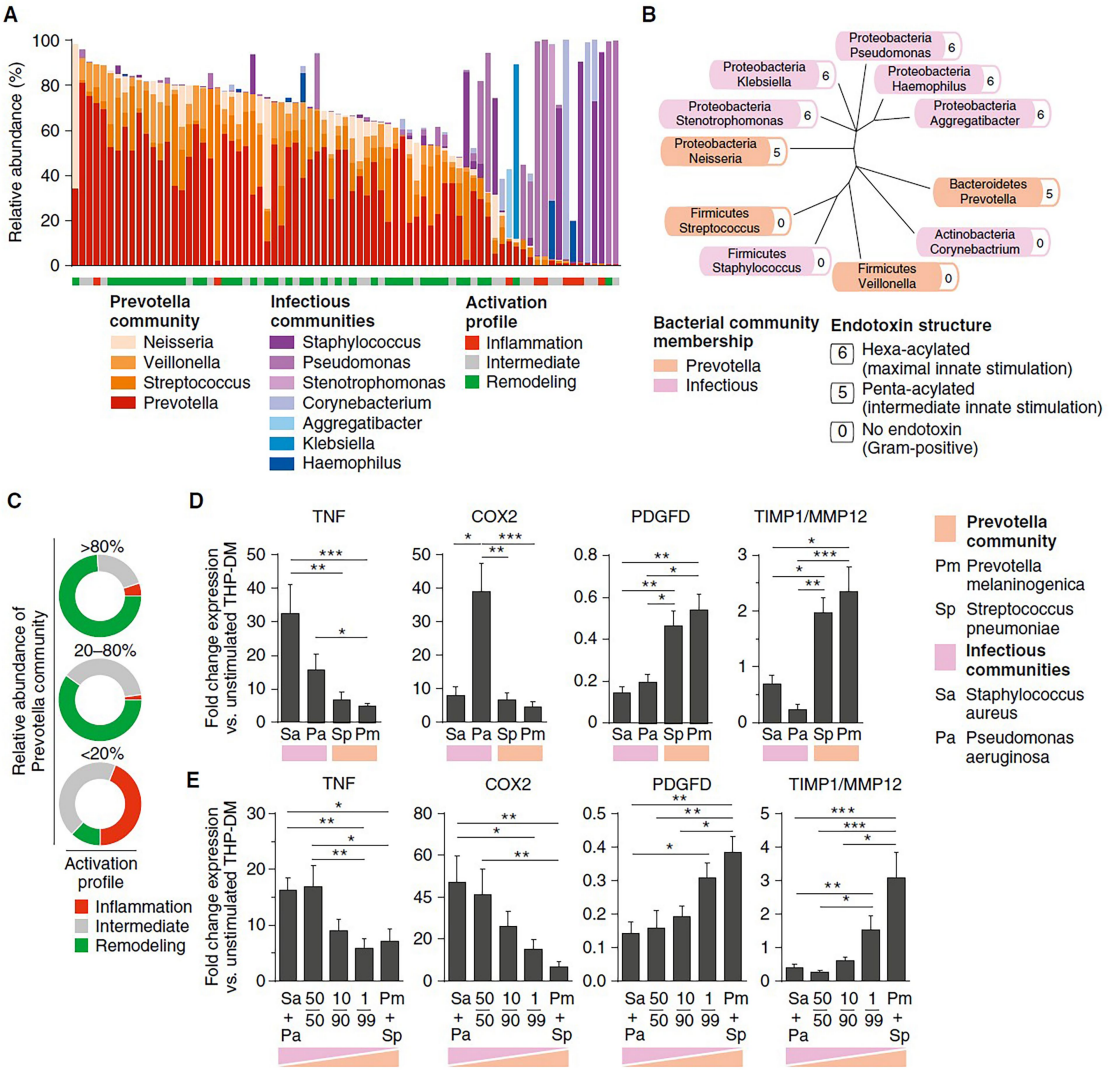
Prominent bacterial genera associated with inflammation, intermediate, or remodeling profiles in a subset of 75 bronchoalveolar lavage fluid samples, and *in vitro* assessment of innate cell activation through various stimuli including reconstituted bacterial communities. **(A)** Relative abundance of bacterial genera derived from 16S sequencing data. Genera are classified as per membership to Prevotella or infectious communities and the underlying innate activation profile is indicated. **(B)** Phylogenetic tree showing phylum assignment of the main bacterial genera, the structure of their endotoxin derived from a previous study (35), and their membership to *Prevotella* or infectious communities (color code). **(C)** Proportion of inflammation, intermediate, and remodeling activation within three sample subsets with decreasing relative abundance of *Prevotella* community. **(D,E)** Quantitative polymerase chain reaction–based gene expression analysis at 18-h post-stimulation enabling quantification of markers of either inflammation (TNF, COX-2) or remodeling (PDGFD, TIMP1/MMP12 ratio), under immunosuppressive conditions. Bacteria belonging to species representative of either infectious communities (*Staphylococcus aureus* and *Pseudomonas aeruginosa*) or *Prevotella* community (*Streptococcus pneumoniae* and *Prevotella melaninogenica*) were incubated with THP-DM at a ratio of 10 colony-forming units per cell. Bacteria of different species were used separately **(D)**, paired based on community membership, or within reconstituted bacterial communities comprising four species at indicated ratios **(E)**. Data were generated in six independent experiments with duplicates or triplicates. Error bars represent SEM. Statistical significance was determined using the Kruskal-Wallis test and Dunn *post-hoc* analysis. *p<0.05, **p<0.01, ***p<0.001. COX, cyclooxygenase; MMP, matrix metalloproteinase; PDGFD, platelet-derived growth factor D; THP-DM, THP-1-derived macrophages; TIMP, tissue inhibitor of metalloproteinase; TNF, tumor necrosis factor, adapted from (18).

The composition of the pulmonary microbiota depends on the immigration of bacteria from the external environment, oral cavity, and upper airways, in addition to the rate of bacterial proliferation and elimination. The transplanted lungs offer special ecologic conditions favorable for microbes (18, 21). In addition to postoperative consequences including poor airway secretion clearance, impaired cough reflex, and a high propensity for micro aspiration, long-term use of immunosuppressive and antibiotic therapy may render local conditions more permissive to certain bacteria otherwise controlled both by the host immunity and competing bacteria in untreated subjects. The Lausanne group assessed whether host microbe interactions in the transplanted lung might determine the immunologic tone of the airways, and consequently could influence graft survival. Microbiota DNA and host total RNA were isolated from 203 bronchoalveolar lavages (BAL) obtained from 112 patients post-lung transplantation issued from SyCLAD cohorts (18). Microbiota composition was determined using 16S ribosomal RNA analysis, and expression of a set of genes involved in prototypic macrophage functions was quantified using real-time quantitative polymerase chain reaction (rt-qPCR). The characteristics of the pulmonary microbiota aligned with distinct innate cell gene expression profiles. Although a nonpolarized activation was associated with bacterial communities consisting of a balance between proinflammatory (e.g., Staphylococcus and Pseudomonas) and low stimulatory (e.g., Prevotella and Streptococcus) bacteria, “inflammatory” and “remodelling” profiles were linked to bacterial dysbiosis. *In vitro* mechanistic assays with bacteria that can be found in the lung have provided direct evidence that bacterial dysbiosis can lead to inflammatory or remodeling patterns in macrophages, whereas balanced bacterial consortia maintained homeostasis, Figure 4. This study was the first to suggest that the pulmonary microbiota impacts long-term graft survival (18).

**Figure 5:**
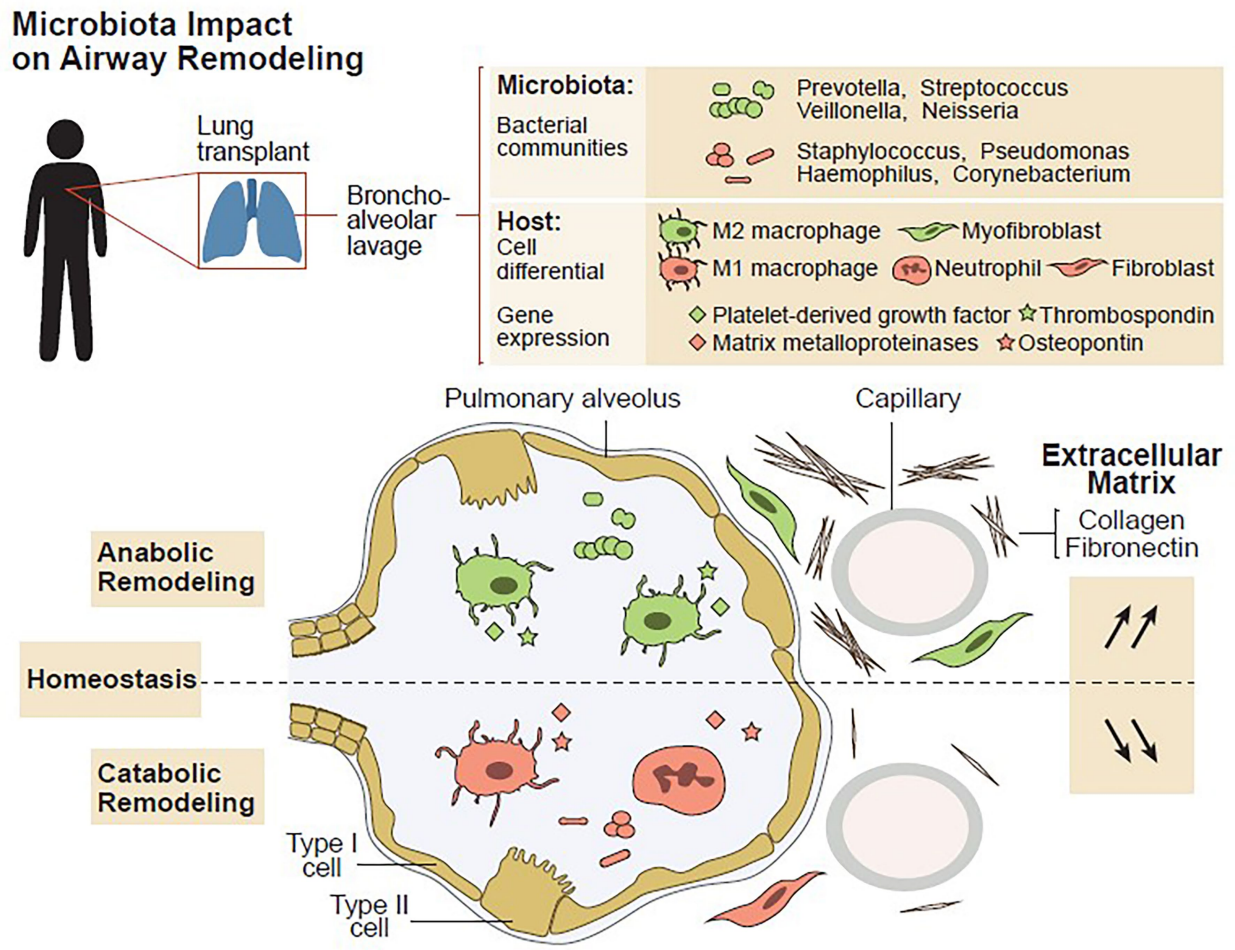
Airway microbiota signals anabolic and catabolic remodeling in the transplanted lung, graphical abstract, adapted from (24).

**Figure 6:**
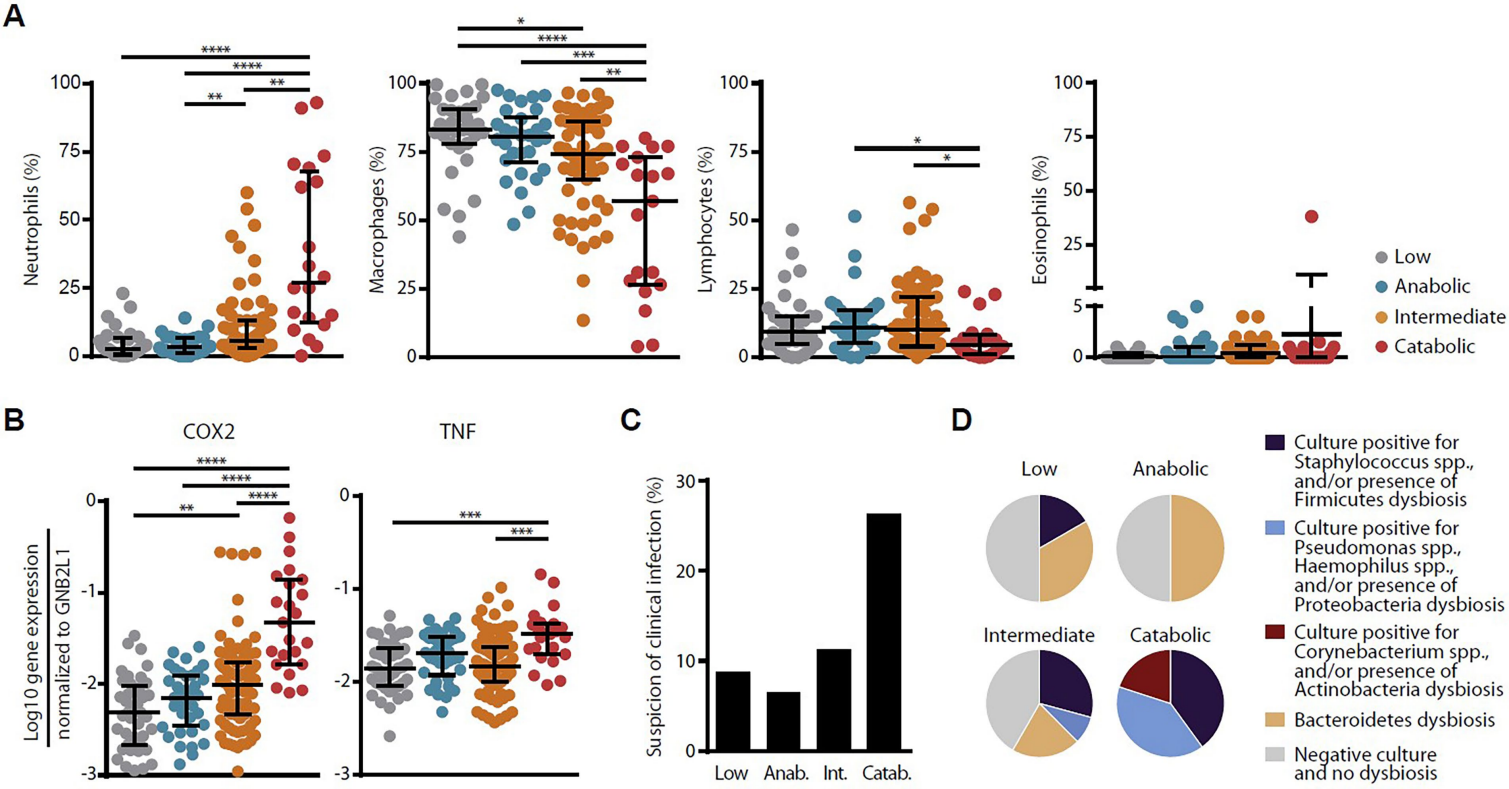
Associations among host remodeling, inflammation, and infection. Relationship among host remodeling and BAL cell differential **(A)**, expression of inflammatory genes COX2 and TNF-a **(B)**, the prevalence of suspected clinical infection **(C)**, and bacteria isolated by culture and/or driving dysbiosis **(D)**. In panels A and B, medians and IQRs are indicated. *p < 0.05, **p < 0.01, ***p < 0.001, and ****p < 0.0001, adapted from (21).

In a subsequent study in Lausanne, host–microbe interactions were dissected in the context of the complex remodeling processes taking place following LT (21). This study assessed whether the local cross talk between the pulmonary microbiota and host cells is a key determinant in the control of lower airway remodeling post-LT, Figure 5. Microbiota DNA and total host RNA were isolated from 189 BAL obtained from 116 patients post LT issued from the SysCLAD cohorts. Expression of a set of 11 genes encoding either matrix components or factors involved in matrix synthesis or degradation (anabolic and catabolic remodeling, respectively) was quantified by RT-qPCR. Microbiota composition was characterized using 16S ribosomal RNA gene sequencing and culture. Four host gene expression profiles were depicted, among which catabolic remodeling, associated with high expression of metallopeptidase −7, −9, and-12, diverged from anabolic remodeling linked to maximal thrombospondin and platelet-derived growth factor D expression. While catabolic remodeling aligned with a microbiota dominated by proinflammatory bacteria (e.g., Staphylococcus, Pseudomonas, and Corynebacterium), anabolic remodeling was linked to typical members of the healthy steady state (eg, Prevotella, Streptococcus, and Veillonella). Mechanistic assays provided direct evidence that these bacteria can affect host macrophage-fibroblast activation and matrix deposition, Figure 6. This study showed that host-microbes interplay potentially determines remodeling activities in the transplanted lung, highlighting new therapeutic opportunities to improve long-term LT outcome.

In a last study, amplicon sequencing of 234 longitudinal BAL samples from 64 LTRs, complemented by bacterial culture to characterize the viable bacterial community established links between the bacterial microbiota, viral loads, and host gene expression in the transplanted lung as well as links with lung function and graft health (32). The lung microbiota post-transplant was categorized into four distinct compositional states, or ‘pneumotypes’. A diverse bacterial community with moderate viral loads and host gene expression profiles suggesting immune tolerance characterizes the predominant ‘balanced’ pneumotype. The other three pneumotypes are characterized by being either microbiota-depleted, or dominated by potential pathogens, and were linked to increased immune activity, lower respiratory function, and increased risks of infection and rejection. Collectively, these findings established a link between the lung microbial ecosystem, human lung function, and clinical stability post-transplant.

### Immunome

#### Activation of dendritic cells

**Figure 7:**
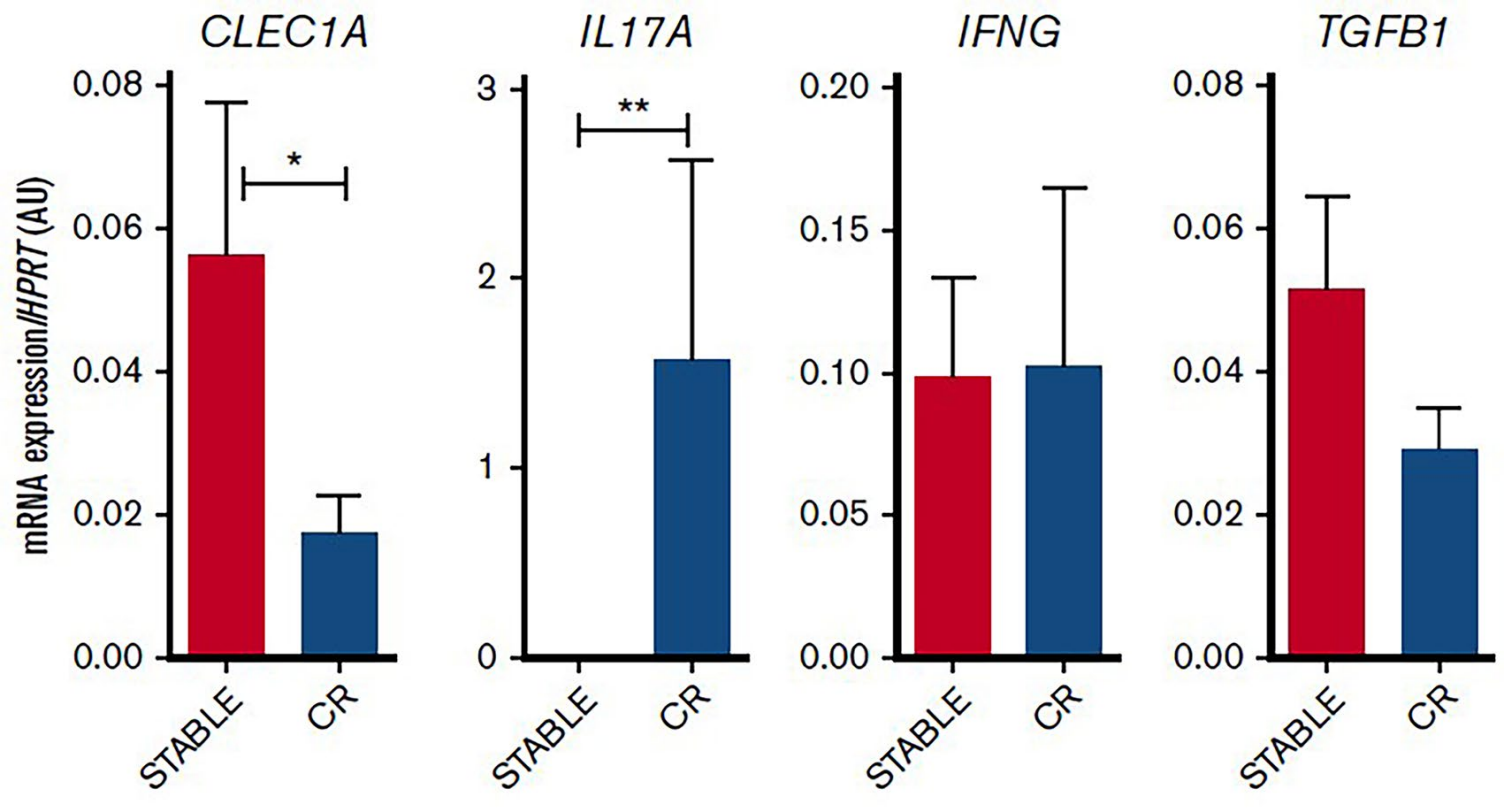
Decreased CLEC1A expression in lung transplants is predictive of CLAD. Lung transplants from stable patients or from patients prior to the development of CLAD were subjected to qRT-PCR for HPRT, CLEC1A, IL17A, IFNG, and TGFB1. Results were expressed in histograms as mean 6SEM of 7 samples in each group and were expressed in AU of specific cytokine/HPRT ratio. *p, 0.05; **p, 0.01. mRNA, messenger RNA, adapted from (19).

Dendritic cells (DCs) represent essential antigen-presenting cells that are critical for linking innate and adaptive immunities, influencing T-cell responses (19). Among pattern recognition receptors, DCs express C-type lectin receptors (CLEC-1) triggered by both exogenous and endogenous ligands, therefore, dictating pathogen response, and also shaping T-cell immunity. Blood was obtained from healthy donors for control. Lung transplant biopsies from stable patients and patients prior to chronic rejection (CR) were obtained from COLT. A decreased expression of CLEC1A in human lung transplants is predictive of the development of chronic rejection and is associated with a higher level of interleukin 17A, Figure 7.

#### Role of T cells to promote the secretion of matrix metalloproteinase-9

**Figure 8:**
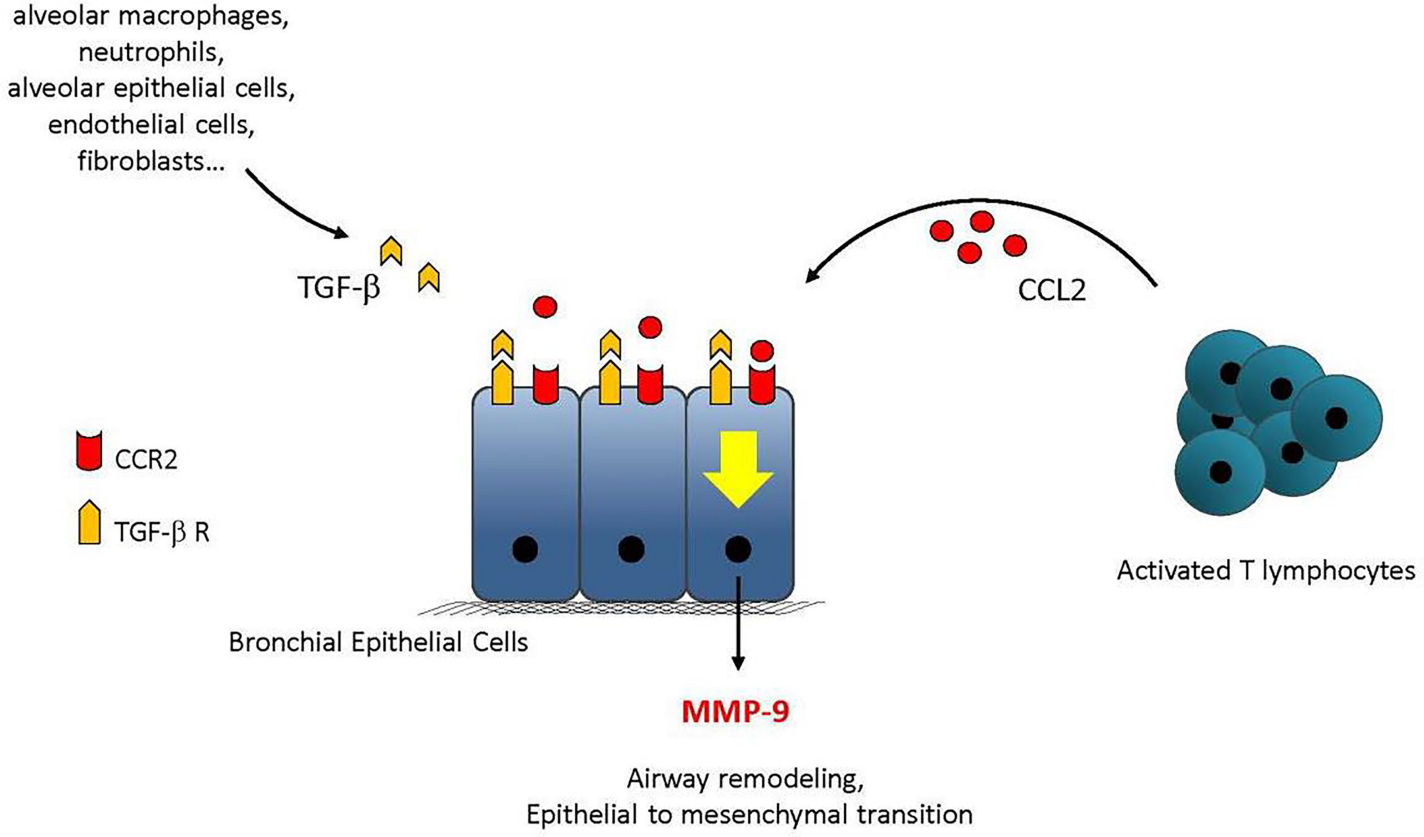
Summary of the main findings. TGF-β can be produced within the graft by alveolar macrophages, neutrophils, endothelial cells, or fibroblasts. CCL2 produced by activated T cells binds to CCR2 and supports the production of MMP-9 in synergy with TGF-β. MMP-9 then contributes to the remodeling processes leading to airway obstruction adapted from (22).

Alloimmune reactions and epithelial-to-mesenchymal transition have been suggested to be involved in BOS (22, 24). However, little was known regarding the role of allogeneicity in epithelial cell differentiation. Primary human bronchial epithelial cells (BECs) were treated with activated T cells in the presence or absence of transforming growth factor (TGF)-β. BECs were obtained from the trachea or bronchi of lung donors from COLT. The expression of epithelial and mesenchymal markers was investigated. The secretion of inflammatory cytokines and matrix metalloproteinase (MMP)-9 was measured in culture supernatants and plasma from LTRs: 49 stable LTRs, 29 with BOS, and 16 with RAS issued from COLT. This study demonstrates that C-C motif chemokine 2 secreted by T cells supports TGF-β-induced MMP-9 production by BECs after binding to C-C chemokine receptor type 2; Figure 8 recapitulates the main results. Longitudinal investigation in LTRs reveals a rise in plasma MMP-9 before CLAD onset. Multivariate analysis shows that plasma MMP-9 is independently associated with BOS (odds ratio [OR] = 6.19, *p* = 0.002) or RAS (OR = 3.9, *p* = 0.024) and predicts the occurrence of CLAD 12 months before the functional diagnosis, Table 2. Thus, immune cells support airway remodeling through the production of MMP-9. Increased plasma MMP-9 is a potential predictive biomarker of CLAD.

**Table 2:** Univariate and multivariate analyses of risk factors for CLAD adapted from (22).

Variable	BOS		RAS	
	*p* value	OR	*p* value	OR
Age	0.996	0.99	0.318	0.16
Sex	0.198	0.54	0.824	1.14
Pathology	0.111	4.37	0.702	1.08
Type of transplant	0.147	0.49	0.269	0.50
Induction	0.358	0.76	0.080	0.51
Azithromycin	0.004	7.06	0.072	3.53
Acute rejection	0.320	0.62	0.130	2.65
LB	0.590	1.74	0.247	3.36
Tacrolimus/Cyclosporin	0.727	0.80	0.858	1.17
Bacteria	0.800	1.21	0.978	0.98
Virus	0.868	0.92	0.216	0.48
Fungi	0.670	0.80	0.119	5.42
MMP-9 (−12)	0.003	4.72	0.024	3.91
MMP-9 (−6)	0.017	3.19	0.300	1.70
MMP-9 (VCLAD)	0.014	4.37	0.007	7.09
Variable	BOS		RAS	
	*p* value	OR (95% CI)	*p* value	OR (95% CI)
Sex	0.036	0.29 (0.08–0.88)		
Azithromycin	0.005	8.14 (2.164–42.018)		
MMP-9 (−12)	0.002	6.19 (2.05–22.33)	0.024	3.90 (1.28–14.05)

Variables with *p*-value inferior to 0.2 in univariate analysis (in bold) were tested in the multivariate analysis. BOS, bronchiolitis obliterans syndrome; RAS, restrictive allograft syndrome; OR, odds ratio; LB, lymphocytic bronchiolitis; MMP, matrix metalloproteinase.

**Figure 9:**
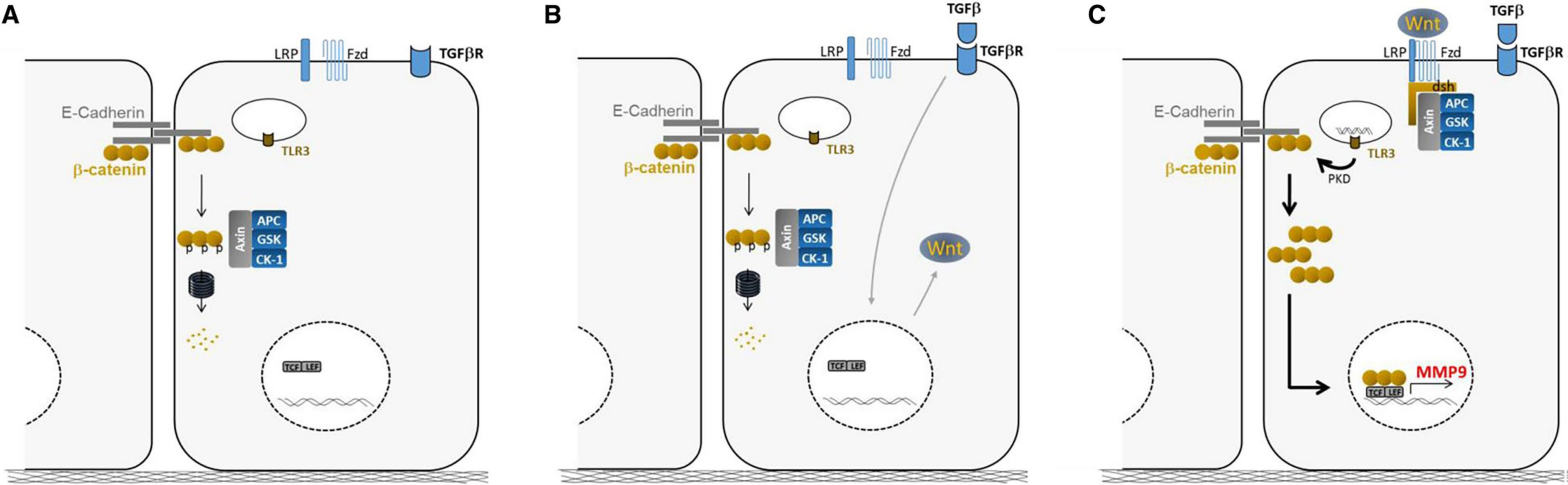
Summary of the main findings. **(A)** Steady-state: cytosolic β-catenin is phosphorylated by the GSK complex and targeted to the proteasome for degradation. **(B)** Wnt ligand production after TGF-β exposure stabilizes the GSK complex at the cell membrane and reduces β-catenin degradation. **(C)** Then, the massive relocation of β-catenin after poly (I: C) treatment, fuels the Wnt/β-catenin pathway and allows β-catenin translocation in the nucleus for MMP-9 expression adapted from (24).

In a mechanistic study, the promotion of MMP-9 was further dissected (24). The response of primary human BEC to viral and bacterial stimulations was investigated in combination with the lung remodeling factor TGF-β. A strong production of pro-inflammatory cytokines by BECs is shown. The production of cytokines and chemokines is dependent on Toll Like Receptor (TLR)-3, except for CXCL10. Mechanistic analyses showed the secretion of Wnt ligands by BEC along with a degradation of the cellular junctions, leading to the release of β-catenin from the cell membrane and stimulation of the Wnt/β-catenin pathway. This study further highlights the cross talk between TGF-β and TLR signaling in bronchial epithelium and its impact on the remodeling process. See Figure 9 for the main results.

#### Blood subpopulation of T and B cells to predict bronchiolitis obliterans syndrome

**Figure 10:**
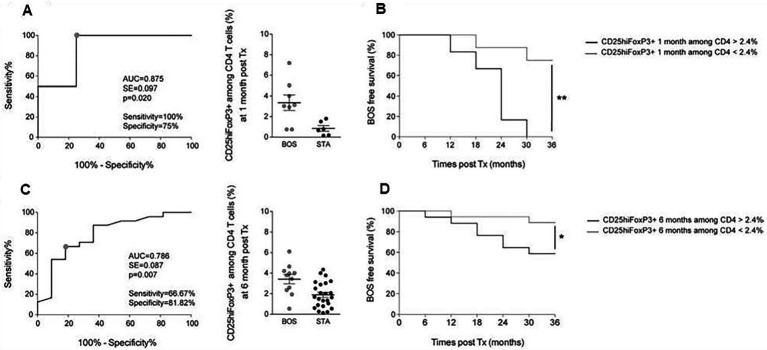
Analysis of CD4^+^CD25^hi^FoxP3^+^ T-cell proportions early post LT as a predictive biomarker of BOS development. **(A)** ROC curve of the proportion of CD4^+^CD25^hi^FoxP3^+^ T cells among CD4 T cells at 1 month after transplantation for BOS (*n* = 8) and STA (*n* = 6). **(B)** Kaplan–Meier analysis of BOS-free survival according to CD4^+^CD25^hi^FoxP3^+^ T-cell proportions among CD4 T cells at 1-month post-transplantation with a cut-off a t2.4% (*n* = 14). **(C)** ROC curve of the proportion of CD4^+^CD25^hi^FoxP3^+^ T cells among CD4 T cells at 6 months post LT for BOS (*n* = 11) and STA (*n* = 24). **(D)** Kaplan–Meier analysis of BOS-free survival according to CD4^+^CD25^hi^FoxP3^+^ T-cell proportions among CD4 T cells at 6 months post-transplantation with a cut-off at 2.4%(*n* = 35). Results are expressed as mean ± SEM. *p < 0.05; **p < 0.01; ***p < 0.001; ****p < 0.0001, adapted from (26).

Blood is a relatively simple window to monitor LTRs (26, 27, 31). Two studies identified T (26) and B (27) cell subpopulations, respectively, as candidates for the early signature of BOS. In-depth profiling of CD4^+^ and CD8^+^ T cells was performed on blood cells from stable (STA) and BOS patients issued form COLT with a longitudinal follow-up. Samples were analyzed at 1 and 6 months after transplantation, at the time of BOS diagnosis, and at an intermediate time-point between 6 and 12 months before BOS diagnosis. Although no significant difference was found for T-cell compartments at BOS diagnosis or several months beforehand, an increase in the CD4^+^CD25^hi^FoxP3^+^ T-cell sub-population was identified in patients with BOS at 1 and 6 months after transplantation (3.39 ± 0.40% vs. 1.67 ± 0.22% in STA, *p* < 0.001). A CD4^+^CD25^hi^FoxP3^+^ T-cell threshold of 2.4% discriminated BOS and stable patients at 1 month post-transplantation, Figure 10. This was validated on a second set of patients at 6 months post-transplantation, Figure 10. Patients with a proportion of CD4^+^CD25^hi^FoxP3^+^ T cells up to 2.4% in the 6 months after transplantation have a 2-fold higher risk of developing BOS. This study is the first to report an increased proportion of circulating CD4^+^CD25^hi^FoxP3^+^ T cells early post-transplantation in recipients who develop BOS within 3 years.

**Figure 11:**
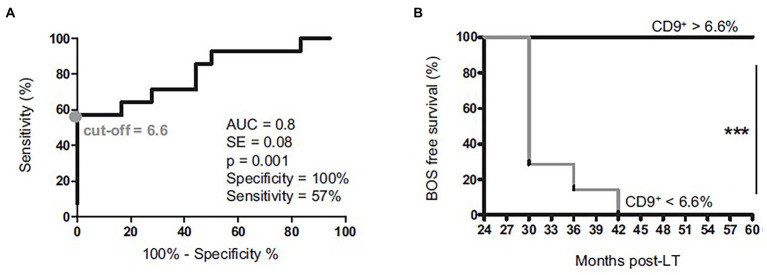
Comparison of graft survival between patients with a % of CD4 + CD57 + ILT2+ T cells (% of CD4+ T cells) ≤ first IQR (25%) at 1-month **(A)** and 6 months **(B)** post-LT vs. those with % CD4 + ILT2 + CD57+ T cells (% of CD4+ T cells) > first IQR (25%) adapted from (27).

B cell profiles were monitored during the early development of BOS after LT (27). The B cell longitudinal profile was analyzed in peripheral blood mononuclear cells from patients with bronchiolitis obliterans syndrome and patients who remained stable over 3 years of follow-up. CD24^hi^CD38^hi^ transitional B cells are increased in stable patients only, and reach a peak 24 months after transplantation, whereas they remain unchanged in patients who developed a BOS. These CD24^hi^CD38^hi^ transitional B cells specifically secrete IL-10 and express CD9. Thus, patients with a total CD9+ B cell frequency below 6.6% display significantly higher incidence of bronchiolitis obliterans syndrome, Figure 11. These data are the first to associate IL-10-secreting CD24^hi^CD38^hi^ transitional B cells expressing CD9 with better allograft outcome in lung transplant recipients. CD9-expressing B cells appear as a contributor to a favorable environment essential for the maintenance of long-term stable graft function and as a potential new predictive biomarker of bronchiolitis obliterans syndrome–free survival.

**Figure 12:**
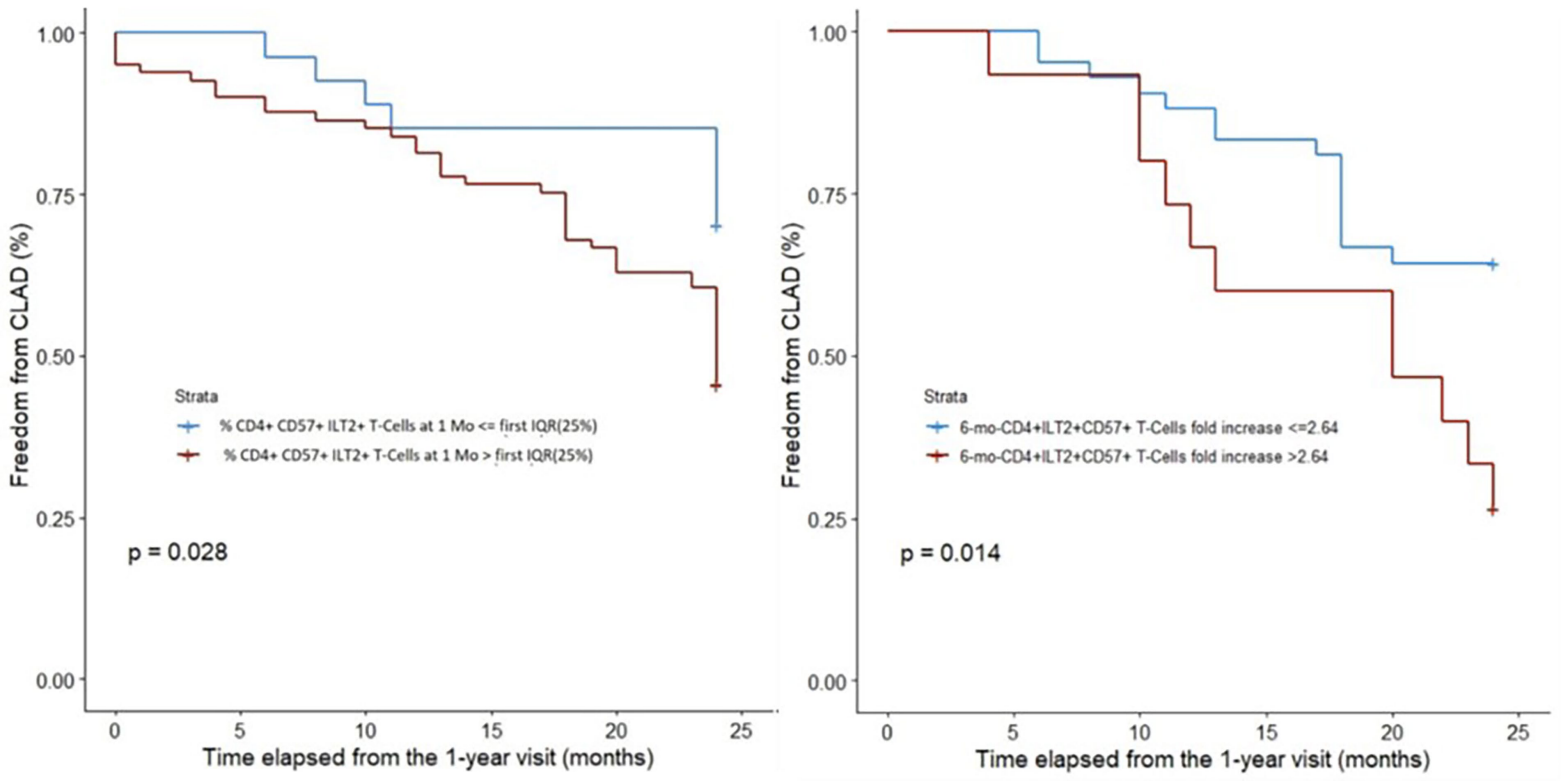
ROC curve and Kaplan–Meier analysis of the level of CD9 + B cells 24 months post-LT in BOS and STA patients of the validation cohort, adapted from (31).

In 2021, the immune checkpoint HLA-G/ILT2 (Ig-Like Transcript 2) expressed by peripheral T-cell subpopulations was investigated to assess whether it could predict CLAD. Data of 150 LTRs from COLT with ≥1 available blood sample at 1-, 6-, or 12 months post-LT were used. Analysis of T cells by flow cytometry focused on the ILT2 receptor of HLA-G and other markers (CD57, CD25, and CD127). T-cell subset analyses compared stable patients and those with CLAD at 3 years post-LT. With data for 78 stable and 72 patients with CLAD, among 21 T-cell subsets expressing ILT2, only CD4 + CD57 + ILT2+ T cells are associated with an outcome. At 1 month post-LT, a low proportion of CD4 + CD57 + ILT2+ T cells is associated with a reduced 3-year incidence of CLAD (CD4 + CD57 + ILT2+ T cells ≤ first IQR [25%] vs. > first IQR, log-rank test, *p* = 0.028). Furthermore, the incidence of CLAD is higher with >2.6-vs. ≤2.6-fold increased proportion of CD4 + CD57 + ILT2+ T cells over the first year post-LT, Figure 12 (3-year freedom frequencies: 27% [95%CI: 8–50] vs. 64% [95%CI: 48–77]) (log-rank test, *p* = 0.014). Upon multivariable analysis, the increased proportion of CD4 + CD57 + ILT2+ T cells over the first year predicted CLAD (hazard ratio 1.25; 95%CI: 1.09–1.44; *p* = 0.001). Focusing on CD4 + CD57 + ILT2+ T cells, this study demonstrates *ex vivo* that these CD4+ T cells are cytotoxic, and selectively inhibited by HLA-G. An early increase of CD4 + CD57 + ILT2+ T cells after LT may be associated with CLAD onset.

### Genome

#### Donor club cell secretory protein G38A polymorphism

**Figure 13:**
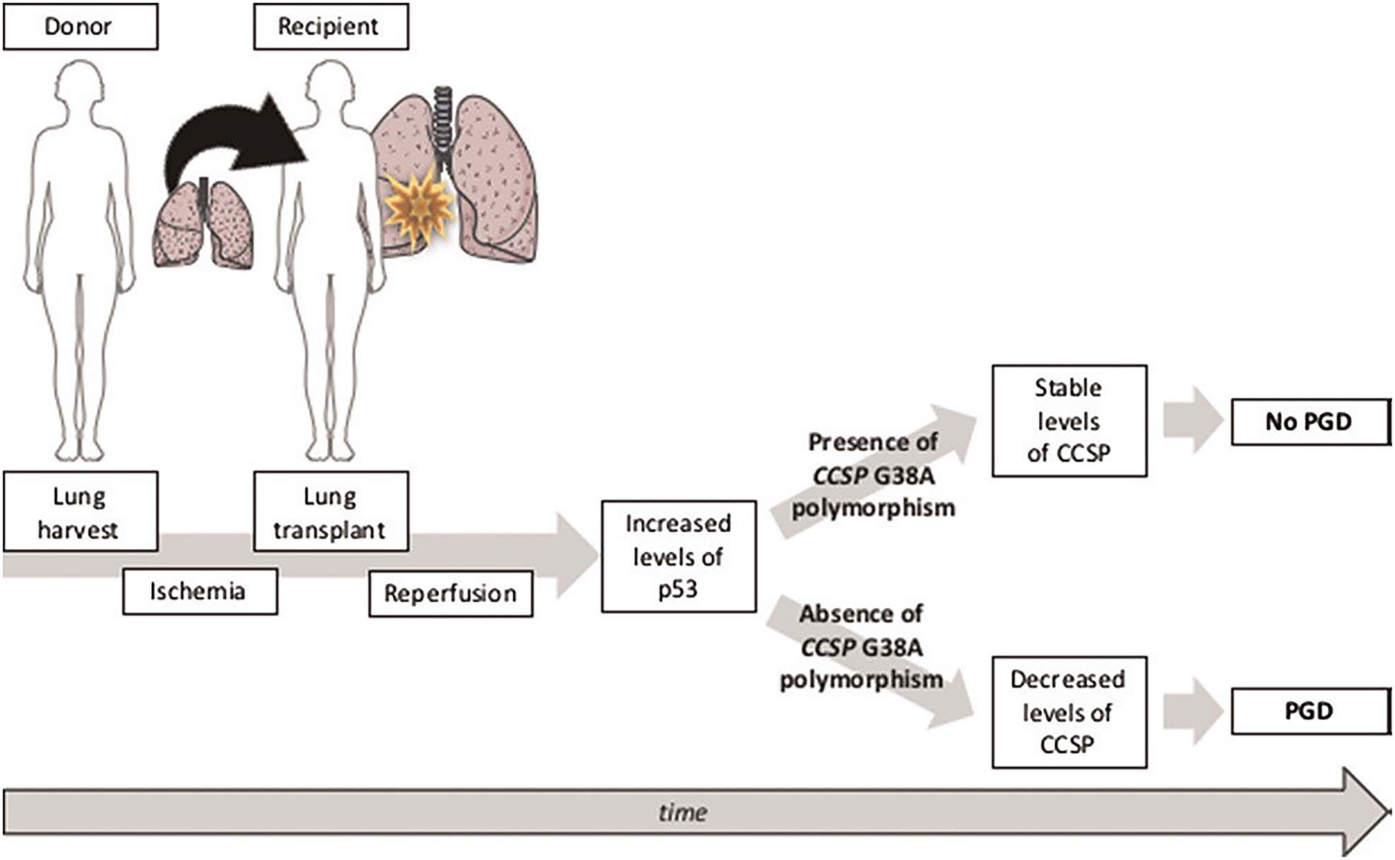
Illustration of the hypothesis: in lung grafts with the G allele of CCSP polymorphism, IR causes an increase in p53 associated with an increase in apoptosis and a decrease in the production of CCSP in lung epithelial cells, thus constituting a vicious circle that may lead to PGD. Conversely, in lung grafts with the A allele of CCSP G38A polymorphism, IR-induced p53 increase has no effect on CCSP expression and CCSP levels, adapted from (29).

Club Cell Secretory Protein (CCSP) G38A polymorphism has recently been involved in lung epithelial susceptibility to external injuries (29). LT is currently limited by ischemia–reperfusion injury leading to primary graft dysfunction (PGD), a recognized risk factor for CLAD. Donor CCSP G38A polymorphism might impact the risk of PGD after LT. Data from the COLT LTRs, performed between January 2009 and December 2014, and associated with preoperative blood samples from the donor and the recipient were collected (29). The CCSP serum concentration and CCSP gene G38A polymorphism were retrospectively determined in a blind manner. Their association with grade 3 PGD was studied in univariate and multivariate analyses. The study group included 104 LT donors and recipients, 84 with grade 0 to 2 PGD and 20 with grade 3 PGD. Preoperative CCSP serum concentration is significantly higher in the donors (median, 22.54 ng/mL; interquartile range, 9.6–43.9) than in the recipients (median, 7.03 ng/mL; interquartile range, 0.89–19.2; *p* < 0.001) but none impact the risk of grade 3 PGD (*p* = 0.93 and *p* = 0.69, respectively). Donor CCSP G38A polymorphism is associated with a decreased risk of grade 3 PGD in univariate (AG + AA 3/21 = 14.2% vs. GG 10/26 = 38.4%, *p* = 0.044) and multivariate analysis (odds ratio associated with AG + AA, 0.22; 95% confidence interval, 0.041–0.88; *p* = 0.045), but recipient CCSP G38A polymorphism is not. Donor CCSP G38A polymorphism is associated with a decreased risk of severe PGD after LT in the COLT study, Figure 13.

### Transcriptome and proteome

**Figure 14:**
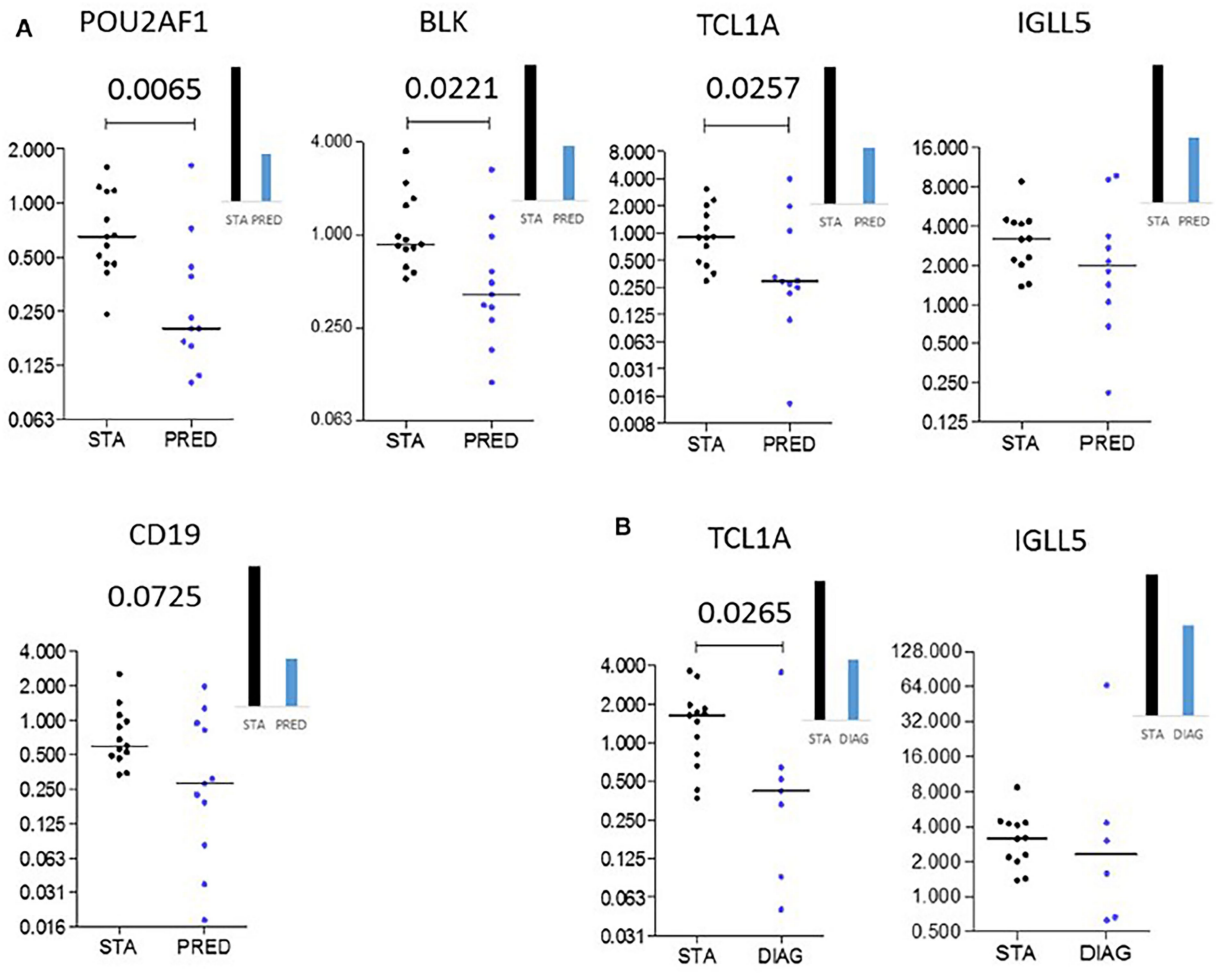
Independent validation. Microarray gene expression data (bar histograms) were validated by quantitative PCR in an independent set of patients (dot histograms) comparing STA and PRED **(A)** and STA and DIAG **(B)**. Mann–Whitney *p* values are indicated, adapted from (25).

**Figure 15:**
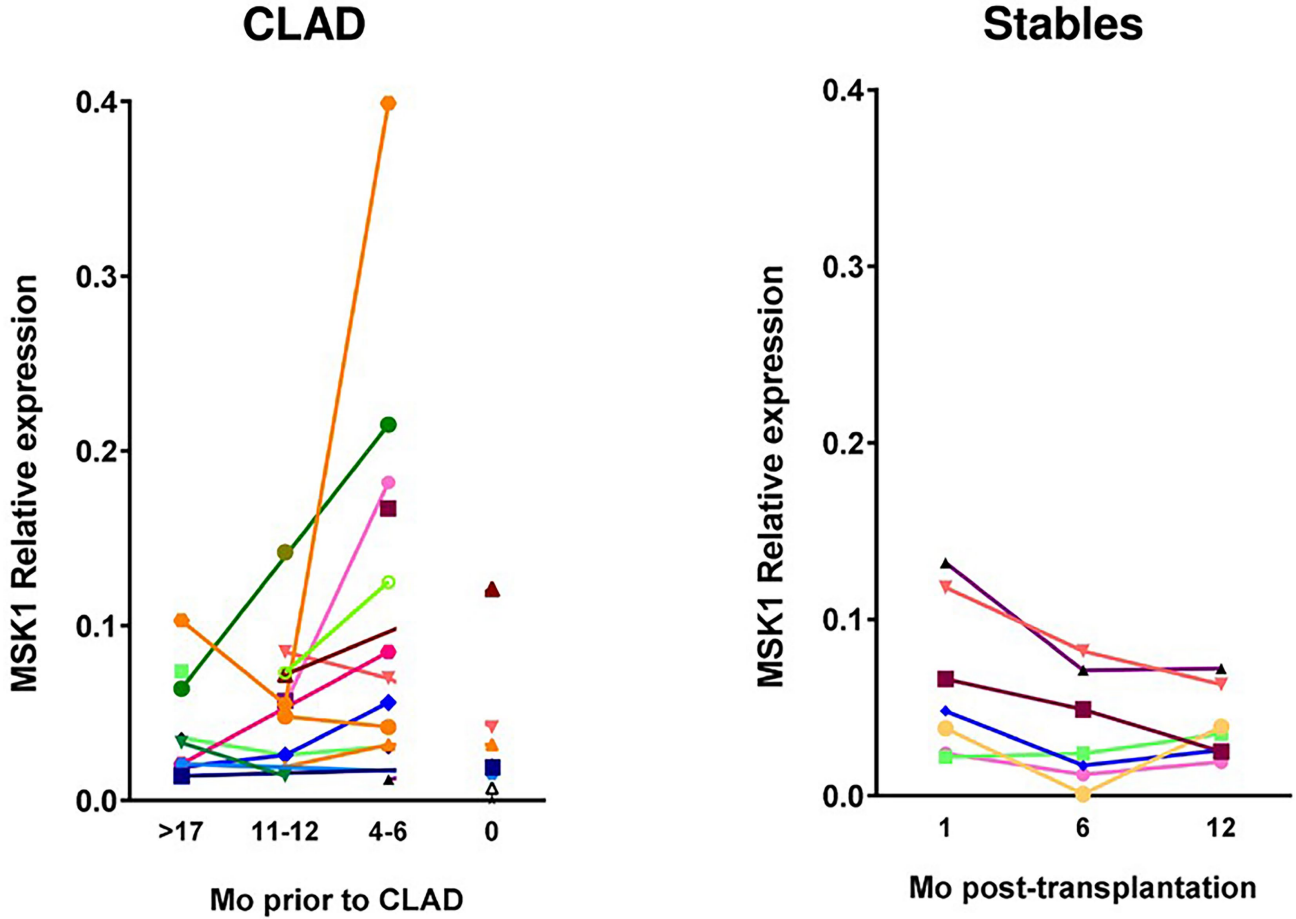
Evolution over time of MSK1 expression in CLAD and stable patients represented by relative RNA expression (1 biopsy per patient and time point). Dots represent value for each patient’s biopsy and line the evolution of expression for each patient over time: for CLAD patients as months before diagnosis, and for stable patients as Mo posttransplantation. CLAD, chronic lung allograft dysfunction; Mo, months; MSK1, mitogen-and stress-activated kinase 1, adapted from (30).

Large-scale gene expression profiling of peripheral blood represents a promising tool for identifying transcriptomic markers associated with the natural history of an allograft (25, 28, 30). By using a large set of 107 blood samples collected in PAXgene tubes from LTRs of COLT, microarray gene expression profiling of whole blood was performed to identify early biomarkers of BOS including samples of 49 patients with stable lung function for at least 3 years, 32 samples collected at least 6 months before BOS diagnosis (prediction group), and 26 samples at or after BOS diagnosis (diagnosis group). An independent set from 25 LTR was used for validation by quantitative PCR (13 stables, 11 in the prediction group, and 8 in the diagnosis group). We identify 50 transcripts differentially expressed between stable and BOS recipients. Three genes, namely POU class 2 associating factor 1 (POU2AF1), T-cell leukemia/lymphoma domain (TCL1A), and B cell lymphocytes, are validated as predictive biomarkers of BOS more than 6 months before diagnosis, with areas under the curve of 0.83, 0.77, and 0.78 respectively, Figure 14. These genes allow stratification based on BOS risk (log-rank test, *p* < 0.01) and are not associated with time post-transplantation (Figure 15).

Acute phase proteins (APPs) may serve as markers that help to control the outcome of transplant recipients (28). Here, we questioned whether plasma concentrations of APPs mirror the development of chronic lung allograft dysfunction (CLAD). We performed a blinded analysis of serial plasma samples retrospectively collected from 35 patients with a lung transplant, of whom 25 developed CLAD and 10 remained stable during the follow-up period of 3–4.5 years. Albumin (ALB), alpha1-antitrypsin (AAT), high sensitivity C-reactive protein (CRPH), antithrombin-3 (AT3), ceruloplasmin (CER), and alpha2macroglobulin (A2MG) were measured by the nephelometric method. We found that within the first 6 months of post-transplantation, levels of A2MG, CER, and AAT are higher in stable patients relative to those who later develop CLAD. Moreover, in stable patients, plasma CRPH levels decreased during the follow-up period as opposed to those developing CLAD in whose CRPH gradually increases. The ALB levels became significantly lower at the end of the follow-up period in CLAD relative to a stable group. A logistic regression model based on A2MG, CER, and AT3 at cut-off levels of ≥175.5 mg/dL, ≥37.8 mg/dl, and ≥ 27.35 mg/dL enables to discriminate between stable and CLAD patients with a sensitivity of 87.5, 100, and 62.5%, and specificity of 65.9, 72.7, and 79.5%, respectively. We identify A2MG (below 175.5 mg/dL) as an independent predictor of CLAD, hazard ratio 11.5, 95% CI (1.5–91.3), *p* < 0.021, Table 3. Our findings suggest that profiles of certain APPs may help to predict the development of lung dysfunction at the very early stages after transplantation.

**Table 3:** Cut-Off levels for APPs at baseline derived by ROC analysis used in single predictor and multivariate logistic models adapted from (28).

Cut-Off at Baseline	Single Predictor Model		Multivariate Model	
	OR (95% CI)	*p* value	OR (95% CI)	*p* value
AT3 ≥ 27.35 mg/dL	10.8 (1.8–64.1)	0.008	13.7 (1.8–107.2)	0.013
A2MG ≥ 175.5 mg/dL	11.5 (1.5–91.3)	0.021	11.5 (1.5–91.3)	0.021
AAT ≥ 169 mg/dL	4.1 (1.4–3.2)	0.073		
CER ≥ 37.8 mg/dL	2.6 (0.96–6.6)	0.010		
Age	1.0 (0.9–1.0)	0.836		
Male sex	1.9 (0.4–8.5)	0.395		

AAT, alpha1-antitrypsin; A2MG, alpha2-macroglobulin; AT3, antithrombin-3; CER, ceruloplasmin; OR, odds ratio; bold highlights statistical significance.

MSK1 expression was assessed in a mouse OB model after heterotopic tracheal allotransplantation (30). Pharmacological inhibition of MSK1 (H89, fasudil, PHA767491) was evaluated in the murine model and in a translational model using human lung primary fibroblasts in proinflammatory conditions. MSK1 expression was graded over time in biopsies from a cohort of patients with CLAD. MSK1 mRNA progressively increases during OB (6.4-fold at D21 post-transplantation). Inhibition of MSK1 allows to counteract the damage to the epithelium (56% restoration for H89) and abolishes the recruitment of MHCII+ (94%) and T cells (100%) at the early inflammatory phase of OB. Finally, we confirm the occurrence of a 2.9-fold increased MSK1 mRNA expression in lung biopsies in patients at 6 months before CLAD diagnosis as compared to recipients with stable lung function. These findings suggest the overall interest of the MSK1 kinase either as a marker or as a potential therapeutic target in lung dysfunction post-transplantation.

**Figure 16:**
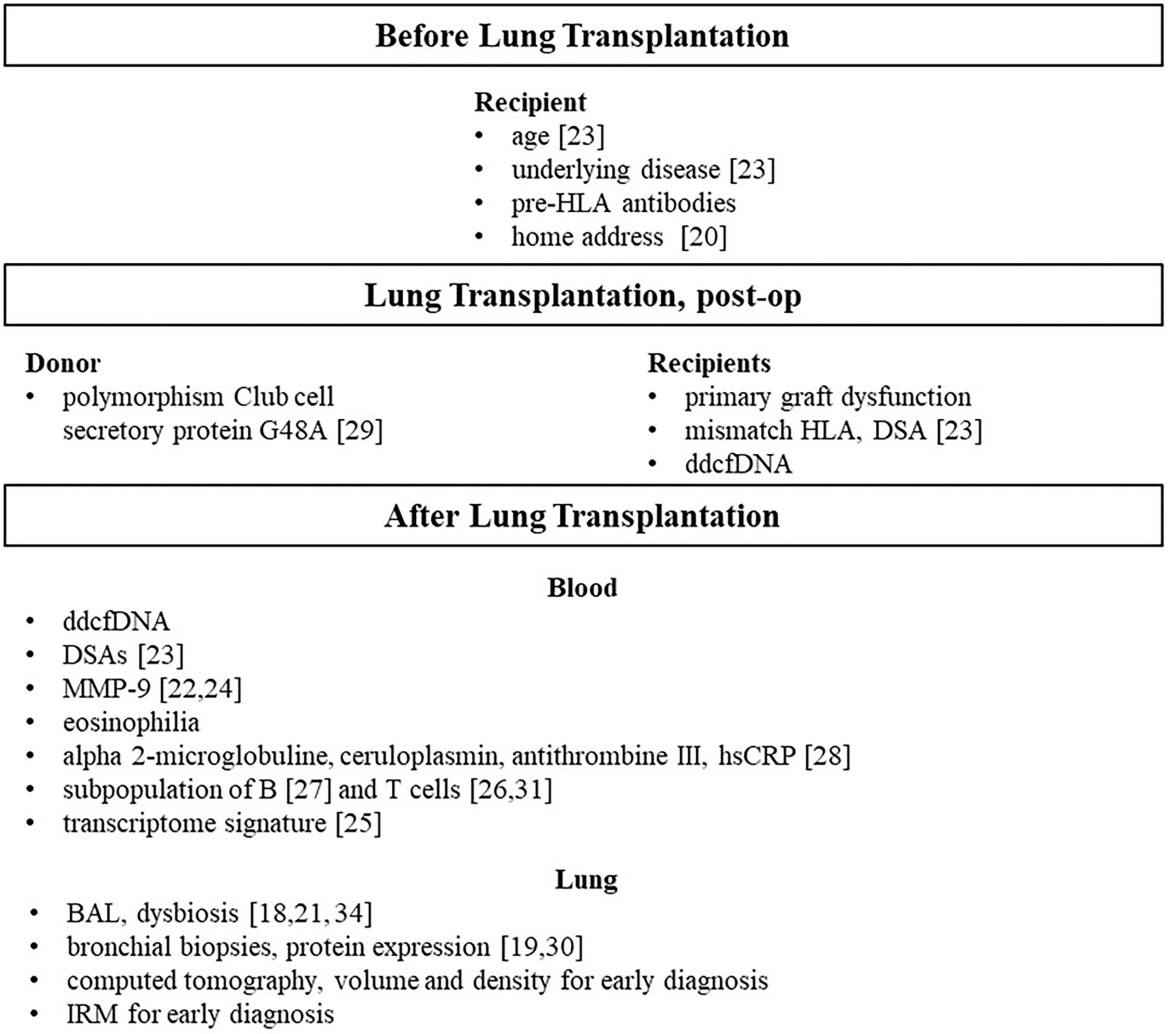
Predictive and/or early marker of CLAD after lung transplantation, main contributions of the COLT and SyCLAD cohorts, reference issued from our cohort studies.

The main results are recapitulated in Figure 16 according to pre, per et post LT area.

## Discussion

An early prediction of CLAD after LT is the main objective of the COLT and SyCLAD cohorts but “*prediction is very difficult, especially about the future*” as stated by Nobel Prize Niels Bohr. Our main results could be discussed as *5 fingerprints* (18–31) in an attempt to get a hand print or signature able to early predict CLAD after LT.

1-Regarding the predictive potential of *clinical risk factors to develop CLAD*, the multivariate prediction model, calibrated within SysCLAD cohorts (23), included recipient age, underlying diagnosis, type of induction treatment, and year-1 class II DSAs. A model, using baseline variables only without DSA had a predictive capacity similar to that of the complete model. The multivariate prediction models for BOS and RAS, respectively, included underlying diagnosis, type of induction treatment, maintenance of immunosuppression, and year-1 class II DSAs, but were not identical. Year-1 class II DSAs are associated with both BOS and RAS with a much stronger weight in the case of RAS, whereas pre-LT diagnoses of ILD/IPF and COPD are associated with RAS. We did not address risk factors related to the donors which may contribute to CLAD through HLA mismatch and primary graft dysfunction issues (36). Independently of year-1 class II DSAs, our finding of an increased risk of RAS in LTRs for COPD and ILD/IPF is new whereas the incidence of BOS is known to be higher in these conditions as compared to LT in cystic fibrosis and was reported within the 2021 ISHLT LT registry (2). In our final model, increasing recipient age has a weak protective effect for CLAD as reported in the 2021 ISHLT registry (2). Evidence is not conclusive concerning induction treatment and the risk for CLAD (37). In 2022, it is now well established that DSAs are a risk factor for BOS and RAS (10). Our ability to predict CLAD early has a major limitation namely the lack of a validation cohort.

2-Because the lung allograft has *constant exposure to the environment and increased susceptibility to infection*, it has a greater risk of rejection compared with other organ transplants. Regarding air pollutants, we (20) and others in the context of LT (38–40) or in the general population (41) have shown strong relationships between emissions due to transport traffic as measured by road length in the buffer zone or distance to the road (39–41), the concentration of fine particulate matter 10 concentration (20, 39, 40) with a lower level in FEV_1_ and FVC (20, 41), and lung function decline (41) with an increased risk of BOS and related-deaths (38–40). Both studies show the protective effects of macrolides on lung function decline after LT (20, 40). A major strength of our study is the analysis of lung function parameters as continuous measurements allowing a more objective assessment of the respiratory health outcome in patients with lung transplant (20). The most striking effect is the time course within few months of air pollutants on lung function in LTRs as compared to the general population suggesting a very high susceptibility of LTRs to any inhaled air contaminants.

Similarly, colonization with a disrupted microbiota or thoracic infection may have direct and indirect effects on the health of the transplanted lung and the risk of CLAD, as shown by three independent sets of experiments on BAL in LTRs conducted by the same group in Lausanne as part of the SysCLAD cohort (18, 21, 32). One of the main breakthroughs of the SysCLAD cohort is the demonstration that lung microbiota is linked to lung function after transplantation. Clinical stability of the graft is linked to the presence of a ‘balanced’ pneumotype characterized by a diverse bacterial community with moderate bacterial and viral loads, and host gene expression profiles suggesting immune tolerance (32).

3-Innate and adaptive immunity closely interact after LT and are modulated by environmental factors and therapeutic intervention as anti-infection and immunosuppressive therapies. Six studies are discussed which successively addressed the activation of dendritic cells (19), secretion of matrix metaloproteinase-9 by activated T cells (22, 24), and three for the blood of T and B subpopulations to predict BOS (26, 27, 31). All these studies explored mechanisms and finally immune biomarkers as candidates are able to predict CLAD after LT (3, 12, 13, 42).

Dendritic cells (DCs) are the sentinels of the immune system that are potentially activated to mediate efficient T-cell priming *via* a set of pattern-recognition receptors (PRRs). Among PRRs, CLEC-1 1 s is shown as a functional cell-surface inhibitory receptor on human DCs that restrains downstream Th17 activation (19). Importantly, decreased expression of CLEC1A in the transplanted lung is shown to predict the Th17-associated development of CLAD (43). Therefore, CLEC-1 may represent a new therapeutic target in a clinical setting to limit Th17 activation and notably lung tissue injury.

Regarding the role of T cells to participate in the epithelial-to-mesenchymal transition (EMT) post LT, a first study (22) investigated in an *ex vivo* model of primary human bronchial epithelial cells (BECs) from lung donor trachea or bronchi the impact of immune cells on the remodeling of the airway epithelium. Experiments were performed in the presence or absence of TGF-β, a growth factor associated with BOS and the foremost inducer of EMT. Activated T cells in synergy with TGF-β promoted specific MMP-9 production by BECs. Collectively these data support the airway epithelium as the main source of MMP-9 and as an active player in CLAD physiopathology. Although MMP-9 has been associated with CLAD, most published results were obtained after BOS onset and therefore had no predictive value. A longitudinal examination was carried out and demonstrates that plasma MMP-9 is independently associated with BOS and RAS 12 months before the diagnosis. One group finds serum MMP-9 (44) and 2 BALF MMP-9 as a potential biomarker of CLAD (45, 46). In a further mechanistic study (24), cross talk between TGF-β and TLR3 signaling favors a remodeling process in the bronchial epithelium. This study supports the airway epithelium as an initiator of immune responses and identifies the Wnt/β-catenin pathway as a potential therapeutic target to tackle the remodeling process associated with CLAD.

Blood subpopulation of T and B cells to predict BOS (26, 27, 31) studies favored the blood window to potentially monitor LTRs. There are numerous immune cells proposed as candidates for CLAD biomarkers, see (12) for review. Among them, we were interested in those that predict or precede CLAD. Regarding the blood T-cell compartment, we are the first to report an increased proportion of circulating CD4 + CD25^hi^FoxP3+ T cells as early as 1 and 6 months post-transplantation in lung recipients who will develop BOS within 3 years. Those CD4^+^CD25^hi^ FoxP3^+^ T cells are mainly characterized as Tregs. It may appear challenging because Tregs are described as potential regulators in an operational tolerance state and are reported to favor good outcomes after solid-organ transplantation. However, a similar cell subset is also present in acute rejection and increases in response to immune activation (47). In accordance with this hypothesis, studies report that inflammatory episodes after transplantation, such as acute rejection (48), ischemia–reperfusion injury, or primary graft dysfunction (49) are risk factors for BOS development. Interestingly, in the context of lung transplantation, Greenland *et al* reported a significant increased proportion of CD4^+^CD25^+^ Tregs in the BAL of patients with the acute rejection of Grade > A1 who will develop BOS (50) suggesting that this increase in Tregs could also be found in the target graft long before BOS development. This point nevertheless remains to be clarified. Since contradictory reports have been published, see (12) for review, early blood monitoring of these cells post-LT is not ready for routine post-LT implementation.

The potential role of the immune checkpoint HLA-G/ILT2 expressed by different *peripheral T-cell subsets* was investigated to predict CLAD onset in LTRs from the COLT cohort (31). An increased proportion of CD4 + CD57 + ILT2+ T cells in a 6-month period within the first year post-LT is associated with CLAD onset at 3 years. The increase is confirmed on multivariate analysis as the only independent predictor of CLAD among all other T-cell subsets analyzed. Phenotyping of CD4 + CD57+ T cells by traditional clues shows that these cells are not senescent with increased density in rejecting allograft tissue, and with high cytolytic properties (51). LTRs without neo-expression of HLA-G in the graft may be more prone to such cytotoxic CD4 + CD57 + ILT2+ T cells insidiously insulting the lung graft, as other senescent T cells associated with BOS (52). Taken together, our results suggest a dysregulated role of HLA-G in LT patients with rejection (53), due to an increased proportion of cytotoxic CD4 + CD57 + ILT2+ T cells, capable of damaging the lung graft without sizeable *in situ* HLA-G graft expression (31).

*B cells* with regulatory properties are associated with the maintenance of long-term allograft function in several transplanted organs (54). The Nantes team and others reported a significant increase in B cells, particularly CD24^hi^CD38^hi^ transitional B cells, in blood from kidney transplanted patients with long-term graft survival in absence of immunosuppression in contrast with patients who reject their graft (55, 56). Regarding B cells and their blood longitudinal monitoring after LT, we report that CD19^+^CD24^hi^CD38^hi^ transitional B cells are correlated with CD9 expression and that the frequency of CD9+ B cells >6.6% at 24 months post-LT allows us to distinguish between patients who will develop BOS within 3 years post-LT and those who will keep a stable graft function (27). Therefore, CD9+ B cell frequency may serve as a predictive biomarker of BOS.

### 4-genome

Donor Club cell secretory protein G38A polymorphism (29) has been associated with a decreased concentration of CCSP in the peripheral blood before LT and with a decreased risk of severe PGD after LT, which is an established risk of CLAD. Several studies (57, 58) suggested that lung ischemia–reperfusion leads to increased levels of CCSP that correlate with altered alveolar epithelial permeability, whereas increased CCSP(+) peripheral blood mononuclear cell mobilization after LT may have beneficial effects on lung oxygenation and recovery time, pointing CCSP and CCSP(+) peripheral blood mononuclear cells as key determinants of early graft function. However, these studies did not consider the genetic variability of donors and recipients over the AG genotype of CCSP. In our study, the AG genotype in the donor is associated with a decreased risk of severe PGD. A tentative explanation might include the resistance of the A allele to p53 (59), a key player in lung ischemia reperfusion. In lung grafts with the CCSP G38A polymorphism, IR-induced p53 increase has no effect on CCSP expression and CCSP levels and is, thus, associated with sustained lung epithelial function (59). This interesting result needs to be replicated in further independent cohorts.

### 5-Transcriptome and proteome

Blood gene expression of a three-gene molecular signature, POU class 2 associating factor 1 (POU2AF1), T-cell leukemia/lymphoma 1A (TCL1A), B lymphoid tyrosine kinase (BLK) differentiating BOS and stable LTRs more than 6 months before diagnosis are identified and have been validated in an independent set (25). To the best of our knowledge, this is the first published study combining two independent cohorts for the identification and validation of predictors of BOS. These three transcripts are biologically plausible as biomarkers of BOS risk after LT since the corresponding gene products are involved in B and T-cell-dependent B cell responses known to be key in tolerance after transplantation. POU2AF1 is a B cell transcriptional coactivator involved in B cell development and function (60). BLK is a member of the Src family of tyrosine kinases and encodes a non-receptor protein tyrosine kinase involved in the regulation of B cell receptor signaling (61). TCL1A is notably expressed by B and T lymphocytes, where it promotes cell proliferation and survival (62).

#### Plasma acute proteins

A logistic regression model based on A2MG, CER, and AT3 measured between 1 and 6 months post LT enabled discrimination between patients who are stable and patients with CLAD. Human A2MG is one of the major blood proteins that bind to a very wide range of substances, including TGF-β1, TNF-α, and IL-1β, and hormones (28). A2MG is also able to inactivate host proteinases, such as trypsin, chymotrypsin, elastase, and metalloproteinases, as well as parasite-derived proteinases. In addition, A2MG binds to iron, zinc, and copper ions in the blood and acts as a serum copper transporter in human blood, thus acting as a host defense against infection. CER is the major copper-carrying protein in the blood, plays a role in iron metabolism, and exhibits glutathione-peroxidase and nitric oxide-oxidase/S-nitrosating activities. CER is also involved in the modulation of coagulation and angiogenesis. Researchers suggest that CER plays a role in tolerance in liver transplantation as a regulator of inflammation and oxidative injury (63). Finally, AT3 is the major inhibitor of proteases. These three proteins are crucial to sustaining the pulmonary defense against inhaled pathogens. Limitations of this study are the small number of patients and the lack of validation.

#### Overexpression of the MSK1 Kinase in patients with CLAD

Besides studies showing MSK1 expression and activation in tracheal allografts model developing OB, MSK1 expression was found constant in patients with stable LT, whereas overexpressed at the 4-to 6-month time point before CLAD diagnosis (30). Further studies, quantifying the activated protein MSK1 in lung biopsies, might help to validate MSK1 as an early marker of CLAD development.

### Strengths

The COLT and SysCLAD cohorts are multi-center and multi-disciplinary and include clinicians, biologists, epidemiologists, SMEs, and systems biologists and have followed prospective designs. To the best of our knowledge, there are few, if any, such consortia that have shared standard operating procedures and goals for more than 10 years in the field of lung transplantation and are still active. National and international lung transplant registries only share clinical data without specific quality controls. (2). The lung allocation score has been shown to be unable to predict death after lung transplantation (64).

Our ambition, partially achieved, was to predict CLAD as early as possible before any decline in lung function and/or clinical manifestations, Figure 16.

With regard to blood biomarkers, in addition to C1Q+ antibodies, HLA-DQ antibodies, and multiple antibodies routinely implanted in long-term care that are associated with an established increased risk of CLAD (10), circulating cell-free DNA (cfDNA) is a promising biomarker. Agbor-Enoh et al. were the first to describe the potential role of repeated measurements of donor-derived cfDNA during the first 3 months after lung transplantation (65). This biomarker at POD 3 has been shown to reflect PGD but also to identify an increased risk of CLAD in a prospective multicentre cohort of 99 LTRs (68). Our main contribution is at the blood level, the easiest monitoring window after LT to predict CLAD. Predictive candidates for CLAD include increased serum MMP-9 levels (22, 24), and a proportion of CD4 + CD25hiFoxP3+ T cells of up to 2.4% within 6 months of LT (69), a frequency of CD9+ B cells below 6.6% with a significantly higher incidence of BOS within 24 months (27) and an increased proportion of CD4 + CD57 + ILT2+ T cells within the first year (31). We also show a promising blood signature of 3 gene transcripts POU2AF1, TCL1A, and BLK that differentiate BOS and stable LTR more than 6 months before diagnosis (25). A validation study PRELUD (PREdiction of Chronic LUng Allograft Dysfunction) is underway in COLT centers (70). Finally, we identify low plasma A2MG levels as a strong and independent predictor of CLAD. (28).

Regarding the environmental risk factors for CLAD, we highlight the deleterious effect of chronic exposure to PM10 (20) and show the predictive value and causal effects of dysbiosis in promoting CLAD after LT (18, 21, 32).

From a clinical point of view, clinical variables and biomarkers for an early predictive signature of CLAD should be easy to collect in a repeatable manner with a focus on the blood window first and BAL if available. In this regard, we propose to calibrate and validate in independent cohorts the best combination of variables including in blood DSA, ddcfDNA, MMP-9, 3 RNA transcripts (POU2AF1, TCL1A and BLK), CD4+CD25hiFoxP3+ % T cells, CD9+ % B cells, CD4+CD57+ILT2+ % T cells and in BAL, microbiota profiles.

### Limitations

Replication has not always been carried out and while we generated many separate fingerprints, only a full fingerprint was offered in 2017 (23). A dynamic full handprint derived from the multiple sources described above, with the potential to predict graft fate, still needs to be calibrated and validated in the future as has been achieved in the context of kidney transplantation (14).

## Perspectives and conclusion

As stated by SE Verleden (13), biomarkers for CLAD before a clinically established diagnosis is much-needed to rapidly predict the future, personalize surveillance and fuel innovative RCTs. In the field of kidney transplantation, the ability to predict chronic dysfunction early (14) has led to an RCT using this proxy to promote early intervention (66) and has the potential to fast track development of pharmaceutical agents. Our LT community would benefit from following this path. The prerequisites are known and have already been successfully applied by the kidney community promoting the iBox score (14–66), *i.e,* multicentre, prospective cohorts, shared SOP and systems approach.

## Members of the Cohort of Lung Transplantation and Systems prediction of Chronic Lung Allograft Dysfunction consortia

Cohort of lung Transplantation-COLT associating surgeons; anaesthetists – intensivists; physicians; research assistants:

Cohort Of Lung Transplantation-COLT (associating surgeons; anaesthetists-intensivists; physicians, research staff) *Bordeaux*: J. Jougon, J. Macey; H. Rozé; E. Blanchard, C. Dromer; X. Demant; *Bruxelles*: M. Ruiz-Patino, M. Vander Kuylen, Y. Sokolow, C. Stefanidis; I. Huybrechts, L. Perrin, L. Van Obberghe, F. Taccone, D. Grimaldi; I. Etienne, C. Knoop, J.L. Vachiéry, C. Dewachter, A. Roussoulières, M. Hites, F. Jacobs; L. Collignon, A-M. Salumu, A Hemelsoet; *Grenoble*: P. Bedouch, A. Briault, Falque, Q. Perrin, C. Pison, C. Saint Raymond; S. Chacaroun, Y. Gioria; *Lyon:* R. Grima, G Drevet, J.-M. Maury, F. Tronc, S. Paulus, P. Portan, J.-F. Mornex, C. Merveilleux du vignaud, E. Chatron, J.-C. Glérant, S. Turquier; D. Gamondes; L. Chalabresse, C. Dubois, A. Rea, M. Reignier, G. Samson; *Paris, Hôpital Européen Georges Pompidou/hôpital Cochin*: N. Carlier, V. Boussaud; F. Le Pimpec-Barthes, A. Bel, P. Achouh, R. Guillemain; *Marseille:* G. Brioude, X.B. D’Journo P. Thomas, D. Trousse; M. Leone, F. Bregeon, B. Coltey, N. Dufeu, A. Nieves; H. Dutau, JY. Gaubert, Ch. Picard, M. Reynaud-Gaubert, D. Boulate, A. Basire, J Bermudez, A. Charvet, B. Coiffard, F. Daviet, JL. Delamarre, JM. Forel, A. Fourdrain, C. Gautier, S. Giusiano, C. Guervilly, P. Habert, S. Hraiech, A. Labarrière, P. Mora, P. Pedini; *Nantes:* P. Lacoste, C. Perigaud, J.C. Roussel, T. Senage, A Mugniot; I. Danner, A Tissot, C Bry, M. Penhouet, E. Eschapasse, D. Horeau-Langlard, FX Blanc; T. Lepoivre, M. Vourch, S. Brouard, R. Danger, M. Bernard, E. Godard, R. Valéro, K. Maugendre, E. Durand, N. Yeremenko, A. Foureau; *Le Plessis Robinson, Hôpital Marie Lannelongue, Paris*: J. Le Pavec, G. Dauriat, P. Pradere, S. Feuillet, S. Dolidon, D. Fabre, E. Fadel, O. Mercier, S. Mussot; D Mitilian, A. Girault, L. Lamrani; Paris, *Hôpital Bichat, Paris*: Y. Castier, P. Mordant, P. Cerceau, A. Roussel; E. Atchade-Thierry, S. Jean-Baptiste, S. Boudinet, A. Gouel, P. Montravers, A. Tran-Dinh, S. Tanaka, N. Zappella, A. Snauwert, P. Tashk, B. Lortat-Jacob; T. Goletto, D. Mouren, M. Salpin, H. Mal, A. Marceau, J. Messika, G. Weisenburger, V. Bunel, L. Genet, S. Trigueiros; A. Bencherif, Y. Costa de Beauregard; *Strasbourg*: P. Falcoz, A. Olland; C.A. Tacquard, G. Ajob, O. Collange, O. Helms, A. Roche; B. Renaud-Picard, R. Kessler, T. Degot, S. Hirschi, A. Schuller, A. Dory, M. Rahli, F. Toti, L. Kessler; J. Stauder; *Suresnes:* A. Chapelier, E.sage, C. Pricopi, M. Glorion, J.De Wolf; M. Le Guen, V. Dumans-Nizard, N. Liu, S. Jacqmin, J. Fessler, M. Davignon, A.Paternot, C. Cerf, AG. Si Larbi, J. Devaquet, G.Tachon, B. Zuber, M. Neuville, E.Cuquemelle, F.Parquin; S. De Miranda, F. Gonin, T.Ngo, D. Usturoi, D. Grenet, A.M. Hamid, C. Picard, A. Roux, O. Brugiére, L.Beaumont-Azuar, S. Colin de Verdière, B. D’Urso, L. Temagoult, C.Bedoui A. Magnan, Q.Marquant, I.Schwartz, H.Salvator; S. Laroche, M. Delahousse, A.Hertig, A.Jalal Eddine, S. Hillaire, F.Mellot, A.Guth, AL. Brun, G.Gravel, E. Longchampt, J.Cohen, M. Vasse, E. Farfour, E.Cardot, E.Joly, Tiffany Pascreau; *Toulouse*: I. Recoche, A. Le Borgne, M. Murris-Espin, P. Rabinel, L. Brouchet, L. Crognier, Olivier Mathe, F. Legenne, M. Barthes, B. Vilquin, A.-L. Costes.

*Swiss lung Transplant centers Lausanne-Geneva:* T. Krueger, H. B. Ris, M. Gonzalez, J.-D. Aubert, L. P. Nicod, B. J. Marsland, C. Berutto, T. Rochat, P. Soccal, Ph. Jolliet, A. Koutsokera, C. Marcucci, O. Manuel, E. Bernasconi, M. Chollet, F. Gronchi, C. Courbon; *Zurich:* S. Hillinger, I. Inci, P. Kestenholz, W. Weder; R. Schuepbach, M. Zalunardo, C. Benden, U. Buergi, L. C. Huber, B. Isenring, M. M. Schuurmans, A. Gaspert, D. Holzmann, N. Müller, T. Rechsteiner, C. Schmid, B. Vrugt. We would like to thank the following collaborators of the Swiss Lung Transplantation Centers for their contribution in data collection and/or project coordination (alphabetical order): E. Catana, C. Cowaloosur-Noirat, M. F. Derkenne, JL Dreifuss, P. Grendelmeier, J. Hartwig, N. Lourenco, M. Magno, H. Muller-McKenna, E. Perret, and K. Zangger.

*Swiss Transplant Cohort Study-STCS:* The members of the Swiss Transplant Cohort Study are: Rita Achermann, Patrizia Amico, John-David Aubert, Philippe Baumann, Guido Beldi, Christian Benden, Christoph Berger, Isabelle Binet, Pierre-Yves Bochud, Elsa Boely, Heiner Bucher, Leo Bühler, Thierry Carell, Emmanuelle Catana, Yves Chalandon, Sabina de Geest, Olivier de Rougemont, Michael Dickenmann, Michel Duchosal, Laure Elkrief, Thomas Fehr, Sylvie Ferrari-Lacraz, Christian Garzoni, Paola Gasche Soccal, Christophe Gaudet, Emiliano Giostra, Déla Golshayan, Karine Hadaya, Jörg Halter, Dominik Heim, Christoph Hess, Sven Hillinger, Hans H. Hirsch, Günther Hofbauer, Uyen Huynh-Do, Franz Immer, Richard Klaghofer, Michael Koller (Head of the data center), Bettina Laesser, Roger Lehmann, Christian Lovis, Oriol Manuel, Hans-Peter Marti, Pierre Yves Martin, Luca Martinolli, Pascal Meylan, (Head, Biological samples management group), Paul Mohacsi, Philippe Morel, Ulrike Mueller, Nicolas J. Mueller (Chairman Scientific Committee), Helen Mueller-McKenna (Head of local data management), Antonia Müller, Thomas Müller, Beat Müllhaupt, David Nadal, Manuel Pascual (Executive office), Jakob Passweg, Juliane Rick, Eddy Roosnek, Anne Rosselet, Silvia Rothlin, Frank Ruschitzka, Urs Schanz, Stefan Schaub, Aurelia Schnyder, Christian Seiler, Susanne Stampf, Jürg Steiger (Head, Executive Office), Guido Stirnimann, Christian Toso, Christian Van Delden (Executive office), Jean-Pierre Venetz, Jean Villard, Madeleine Wick (STCS coordinator), Markus Wilhelm, and Patrick Yerly.

*SME and Platforms Biomax (Munich, Germany):* A. Fritz, D. Maier; *Finovatis (Lyon, France):* K. Desplanche, D. Koubi; *GATC* (*Germany):* F. Ernst, T. Paprotka, M. Schmitt, B. Wahl; *Novasdicovery (Lyon, France):* J.-P. Boissel, G. Olivera-Botello; *Prométhée Proteomics Platform (Grenoble, France):* C. Trocmé, B. Toussaint, S. Bourgoin-Voillard, M. Séve; *Inserm U823, Université GrenobleAlpes (Grenoble, France):* M. Benmerad, V. Siroux, R. Slama; *European Institute for Systems Biology & Medicine (Lyon, France):* C. Auffray, B. de Meulder, D. Lefaudeux, J. Pellet.

## Author contributions

CP drafted the manuscript. All authors performed data collection and harmonization, participated in the adjudication committee, and revised the manuscript for important intellectual content.

## Funding

The SysCLAD study is an EU-funded project, HEALTH-F5-2012 (grant agreement #305457) under the Seventh Framework Programme (FP7). The authors are indebted to the “Programme Hospitalier de Recherche Clinique 2008,” “Vaincre la Mucoviscidose,” and Association Grégory Lemarchal for supporting this project from its beginning, when 11 French lung transplantation centers gave rise to the Cohort in Lung Transplantation (COLT), “Programme Transplantation 2008,” PRTP-13, http://ClinicalTrials.gov Identifier: NCT00980967. This work was realized in the context of the IHU-Cesti project thanks to the French government’s financial support managed by the National Research Agency *via* the “Investment into the Future” program ANR-10-IBHU-005. The IHU-Cesti project is also supported by Nantes Métropole and Région Pays de la Loire. The authors thank the Institut de recherche en santé respiratoire des pays de Loire for their financial support. This study has been conducted in the framework of the Swiss Transplant Cohort Study, supported by the Swiss National Science Foundation and the Swiss University Hospitals (G15) and transplant centers. The authors thank the Swiss National Research Foundation for supporting the STCS and in particular the lung transplant section (No 3347CO-108795) and the Juchum foundation. The funders had no role in study design, data collection and analysis, decision to publish, or preparation of the manuscript.

## Conflict of interest

The authors declare that the research was conducted in the absence of any commercial or financial relationships that could be construed as a potential conflict of interest.

## Publisher’s note

All claims expressed in this article are solely those of the authors and do not necessarily represent those of their affiliated organizations, or those of the publisher, the editors and the reviewers. Any product that may be evaluated in this article, or claim that may be made by its manufacturer, is not guaranteed or endorsed by the publisher.

## Abbreviations

AUC: Area under the curve
BMI: body mass index
BAL: bronchoalveolar lavage
BOS: bronchiolitis obliterans syndrome
CLAD: chronic lung allograft dysfunction
COLT: cohort of lung transplantation
DSAs: donor specific antibodies
FEV_1_: forced expiratory volume in one second
FVC: forced vital capacity
HLA: human leukocyte antigen
ISHLT: International society for heart and lung transplantation
ILD/IPF: interstitial lung disease, idiopathic pulmonary fibrosis
LRA: logistic regression analysis
LT: lung transplantation
LTR: lung transplantation recipient
PFT: pulmonary function tests
PGD: primary graft dysfunction
OR: odds ratio
RAS: restrictive allograft syndrome
rATG: rabbit anti-thymocyte globulin
ROC: receiver operating characteristic
SysCLAD: Systems prediction of Chronic Lung Allograft Dysfunction
t-AR: treated acute rejections
t-CMV: treated CMV infections
t-infections: treated infections
TLC: total lung capacity
